# Pulmonary circulation-mediated heart targeting for the prevention of heart failure by inhalation of intrinsically bioactive nanoparticles

**DOI:** 10.7150/thno.61875

**Published:** 2021-07-25

**Authors:** Chao Liu, Liyuan Chen, Yongchang Ma, Kaiyao Hu, Peng Wu, Lina Pan, Haiyan Chen, Lanlan Li, Houyuan Hu, Jianxiang Zhang

**Affiliations:** 1 Department of Cardiology, Southwest Hospital, Third Military Medical University (Army Medical University), Chongqing 400038, China; 2 Department of Pharmaceutics, College of Pharmacy, Third Military Medical University (Army Medical University), Chongqing 400038, China; 3 State Key Lab of Trauma, Burn and Combined Injury, Institute of Combined Injury, Third Military Medical University (Army Medical University), Chongqing 400038, China

**Keywords:** heart failure, cardiac dysfunction, bioactive nanoparticles, nanotherapy, inhalation delivery, targeted therapy

## Abstract

Heart failure is a serious clinical and public health problem. Currently there is an unmet demand for effective therapies for heart failure. Herein we reported noninvasive inhalation delivery of nanotherapies to prevent heart failure.

**Methods:** A reactive oxygen species (ROS)-scavenging material (TPCD) was synthesized, which was processed into antioxidative and anti-inflammatory nanoparticles (*i.e.*, TPCD NP). By decoration with a mitochondrial-targeting moiety, a multilevel targeting nanotherapy TTPCD NP was engineered. Pulmonary accumulation of inhaled TPCD NP and underlying mechanisms were examined in mice. *In vivo* efficacies of nanotherapies were evaluated in mice with doxorubicin (DOX)-induced cardiomyopathy. Further, an antioxidative, anti-inflammatory, and pro-resolving nanotherapy (*i.e.*, ATTPCD NP) was developed, by packaging a peptide Ac2-26.* In vitro* and *in vivo* efficacies of ATTPCD NP were also evaluated.

**Results:** TPCD NP alleviated DOX-induced oxidative stress and cell injury by internalization in cardiomyocytes and scavenging overproduced ROS. Inhaled TPCD NP can accumulate in the heart of mice by transport across the lung epithelial and endothelial barriers. Correspondingly, inhaled TPCD NP effectively inhibited DOX-induced heart failure in mice. TTPCD NP showed considerably enhanced heart targeting capability, cellular uptake efficiency, and mitochondrial localization capacity, thereby potentiating therapeutic effects. Notably, TPCD NP can serve as bioactive and ROS-responsive nanovehicles to achieve combination therapy with Ac2-26, affording further enhanced efficacies. Importantly, inhaled TPCD NP displayed good safety at a dose 5-fold higher than the efficacious dose.

**Conclusions:** Inhalation delivery of nanoparticles is an effective, safe, and noninvasive strategy for targeted treatment of heart diseases. TPCD NP-based nanotherapies are promising drugs for heart failure and other acute/chronic heart diseases associated with oxidative stress.

## Introduction

Heart failure is a leading cause of morbidity, mortality, and hospitalizations in developed countries. In the United States, approximately 6.2 million adults had heart failure, while over 26 million patients with heart failure worldwide 1-3. The 5-year mortality rate of heart failure patients is about 50%, despite recent advances in prevention and therapy of heart failure over the past decades 4. In view of the crucial role of myocardial oxidative stress in the development and progression of heart failure, different antioxidants have been investigated in both preclinical and clinical studies for the treatment of heart failure, by regulating mitochondrial reactive oxygen species (ROS) generation, scavenging excessive ROS, or attenuating oxidative damage 5. Unfortunately, most current strategies have failed to provide beneficial clinical outcomes 6. To a large degree, this can be attributed to the low accumulation and short retention time of therapeutic molecules in the heart, adverse effects resulting from nonspecific drug distribution and overdose treatments, and/or limited antioxidative activity of available drugs. Accordingly, both new therapies and novel delivery approaches are necessary for the treatment of heart failure.

Recently, increasing evidence has demonstrated advantages of nanoparticle (NP)-based targeting strategies in the treatment of heart diseases 7-13, such as heart failure, myocardial infarction, hypertrophic cardiomyopathy, and other ischemic heart diseases. In this aspect, different types of NPs, such as polymeric NPs 14, liposomes 15, inorganic nanovehicles 16, and biomimetic/bioinspired NPs 17, have been examined for site-specific delivery of different therapeutic agents to the heart 18-23. In addition to passive targeting after systemic administration, heart accumulation efficiency can be further enhanced by surface engineering of NPs with functional molecules or specific cellular membranes 18, 21, 22, 24. However, most currently developed nanotherapies have been investigated for the treatment of heart diseases by administration via the intravenous, intracoronary, intrapericardial, or intramyocardial injection. These delivery approaches exhibit multiple disadvantages, such as invasiveness, complicated operation procedures, difficulty in accurately locating injection sites, and causing additional side effects, thereby affording poor patient compliance and adherence. This is especially true for the long-term treatment of chronic heart diseases. Consequently, effective and safe delivery strategies remain to be established for facile and translational heart targeting of NPs.

As well documented, inhaled microparticles, especially ultrafine particles with diameters < 100 nm, can be transported from the lungs to blood circulation through the air-blood barrier 25-31, which is closely related to endothelial injury and systemic inflammation resulting from air pollution 32-34. Moreover, the heart accumulation of inhaled NPs was also observed 19, 35, indicating that inhalation delivery is promising for the treatment of heart diseases. As a noninvasive route, inhalation delivery is frequently employed in the treatment of respiratory diseases, due to a number of advantages such as easy handling, self-administration, good patient compliance and adherence, rapid absorption/action, low metabolism, and reduced risk of systemic adverse effects 36-40. In particular, the lungs have large surface area for absorption, a thin cell barrier, good epithelial permeability, extensive vascularization, and large blood flow. All these characteristics are beneficial to improve the bioavailability of inhaled drugs and facilitate their transport to the bloodstream 41. Recently, a growing number of studies have suggested that inhalation delivery is an intriguing approach for noninvasive systemic delivery of drugs through the lungs for the treatment of autoimmune and cardiovascular diseases 19, 25, 36, 40, 42. Despite the great promise of targeted therapy of heart diseases by inhalation of nanotherapies, the mechanisms underlying pulmonary translocation and heart targeting of inhaled NPs remain elusive.

**Figure 1:**
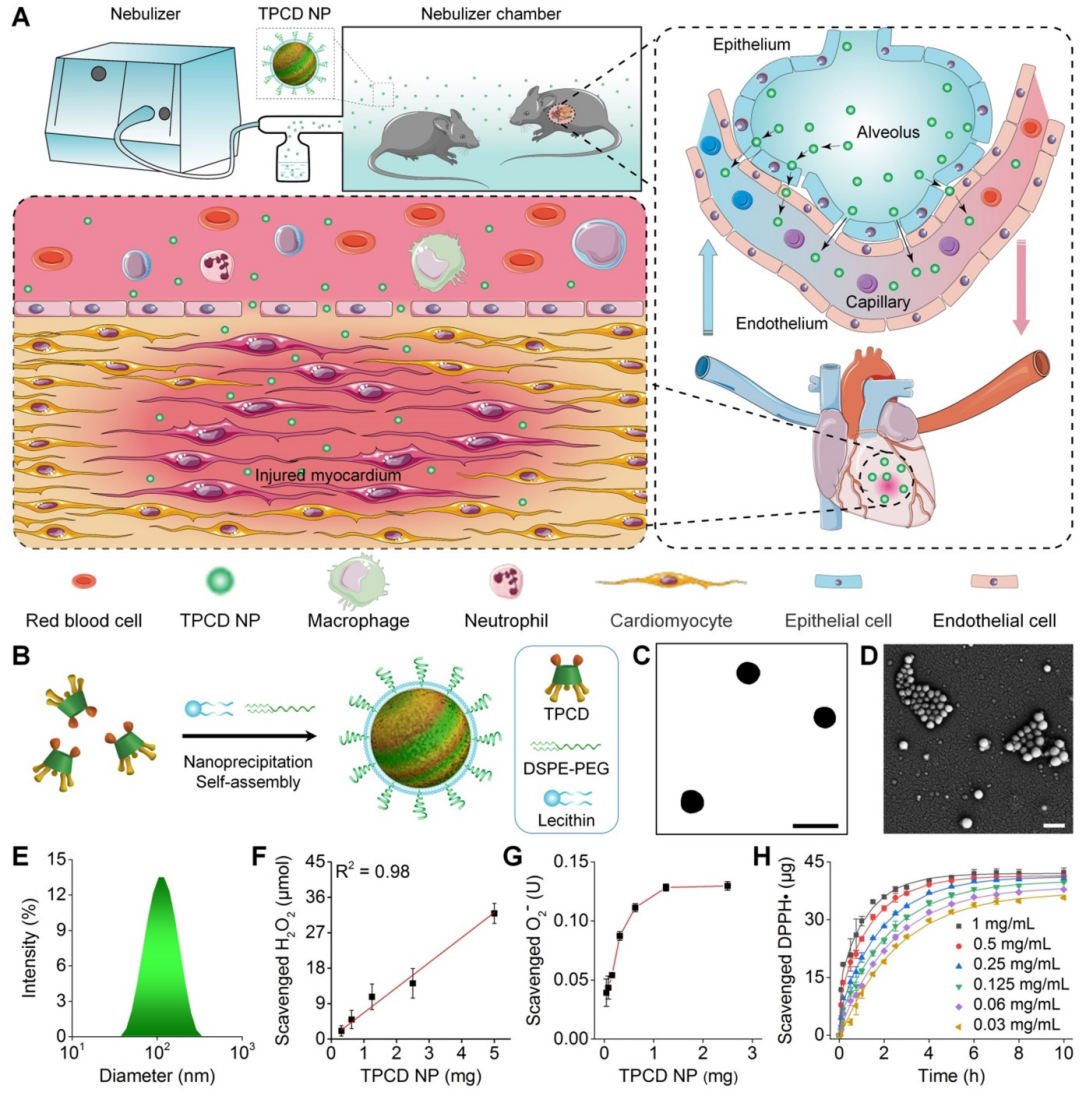
Pulmonary circulation-mediated targeted therapy of heart failure by inhalation of bioactive nanotherapies. (A) Schematic illustration of *in vivo* targeting of the injured myocardium via inhalation of a nanotherapy TPCD NP. (B) A sketch showing preparation of a bioactive nanotherapy by nanoprecipitation/self-assembly. (C-E) Typical TEM (C) and SEM (D) images as well as size distribution (E) of TPCD NP. (F-H) Dose-dependent elimination of H_2_O_2_ (F) and superoxide anion (G) as well as time- and dose-dependent scavenging of DPPH radical (H) by TPCD NP. Scale bars in (C-D): 200 nm. Data in (F-H) are mean ± SD (n = 3).

Here we hypothesize that NPs deposited in the lungs can be gradually and continuously transported to the heart by the pulmonary circulation (Figure 1A). As a proof of concept, a nanotherapy derived from antioxidative NPs was employed in this study, considering the critical role of oxidative stress in heart failure. We found time-dependent translocation of inhaled NPs via the pulmonary epithelial and endothelial cells, with subsequent heart accumulation through the pulmonary circulation via the coronary arteries in mice, thereby affording good therapeutic effects in a mouse model of heart failure. In addition, we speculate that antioxidative stress in combination with anti-inflammation can afford more potent therapeutic effects, in view of the fact that inflammation plays an important role in the pathogenesis of acute and chronic heart failure 43, 44. To demonstrate this hypothesis, Ac2-26 was examined as a model anti-inflammatory agent. Notably, Ac2-26 is an annexin A1 N-terminal derived peptide with pharmacological activities of this endogenous proresolving protein 45. Previous studies have demonstrated that Ac2-26 can inhibit the infiltration of neutrophils and monocytes/macrophages as well as provide tissue protective effects in animal models peritonitis and atherosclerosis 46-48.

## Methods

### Materials synthesis and characterization

A ROS-scavenging material (defined as TPCD) was synthesized by sequentially conjugating Tempol (Tpl) and 4-(hydroxymethyl)phenylboronic acid pinacol ester (PBAP) onto β-cyclodextrin (β-CD) 49. Stearyl triphenylphosphine (TPP) (*i.e.*, STPP) was synthesized according to the previously reported method 50. Fourier transform infrared (FT-IR) spectra were recorded on a PerkinElmer FT-IR spectrometer (100S). ^1^H NMR spectroscopy was performed by an Agilent DD2 600 MHz NMR spectrometer.

### Preparation of TPCD nanoparticles and TPP-decorated TPCD nanoparticles

TPCD nanoparticles (TPCD NP) were prepared according to our previously established method 51. Briefly, 3 mg lecithin and 4.5 mg 1,2-distearoyl-sn-glycero-3-phosphoethanolamine-N-[methoxy(polyethylene glycol)-2000] (DSPE-PEG) were dissolved in 0.3 mL of ethanol, which was added to 7.5 mL of deionized water. The obtained mixture was stirred at 65°C for 1 h to give rise to an aqueous phase, into which 25 mg TPCD dissolved in 2.5 mL of methanol was added. After stirring at room temperature for 2 h, TPCD NP was obtained by removing the organic solvents and excess distilled water via rotary evaporation. Through similar procedures, NPs labeled with cyanine5 NHS ester (Cy5) or cyanine7.5 NHS ester (Cy7.5) were prepared, which were termed as Cy5/TPCD NP and Cy7.5/TPCD NP, respectively.

To prepare TPP-decorated TPCD NPs (TTPCD NP), lecithin (3 mg), DSPE-PEG (4.5 mg), and STPP (3.75 mg) were dissolved in 0.45 mL of ethanol. Thus formed solution was mixed with 7.5 mL of deionized water, and the mixture was stirred at 65°C for 1 h to obtain an aqeuous phase. Then TPCD (25 mg) dissolved in methanol (2.5 mL) was added to the aqueous phase, followed by similar procedures as aforementioned to collect TTPCD NP.

### Preparation of TPCD nanoparticles containing Ac2-26

Lecithin (3 mg) and DSPE-PEG (4.5 mg) were dissolved in 0.45 mL of ethanol, which was mixed with 7.5 mL of deionized water and stirred at 65°C for 1 h to obtain an aqueous phase. TPCD (25 mg) was dissolved in 2.5 mL of methanol, into which 50 μg Ac2-26 dissolved in 50 μL of DMSO was added. The obtained organic solution was added into the aqueous phase. After magnetic stirring at room temperature for 2 h, the solution was centrifuged at 5000*g* for 10 min to collect ATPCD NP. Following similar procedures, TPCD NPs containing FITC-labeled Ac2-26 (FITC-ATPCD NP) and TPP-decorated TPCD NPs containing Ac2-26 (ATTPCD NP) were fabricated.

### Quantification of Ac2-26 contents in TPCD nanoparticles

To quantify the loading content of FITC-Ac2-26, different TPCD NPs were dissolved in methanol. Then fluorescence intensities of FITC-Ac2-26 were measured by fluorescence spectroscopy (F-7000, Hitachi, Japan), with excitation wavelength at 494 nm and emission wavelength at 518 nm.

### *In vitro* release of Ac2-26 from TPCD nanoparticles

Specifically, 40 mL of PBS (0.01 M, pH 7.4) containing 5 mg FITC-ATPCD NP with or without 1 mM H_2_O_2_ was separately incubated at 37°C. At predetermined time intervals, 1 mL of the medium was withdrawn and the same volume of fresh medium was supplemented. After centrifugation of the collected medium at 20000*g* for 15 min, the supernatant was collected and the concentration of Ac2-26 was determined by fluorescence spectrophotometry.

### Cell culture

H9C2 rat cardiomyoblast cells, A549 human lung alveolar cells, human umbilical vein endothelial cells (HUVECs), and RAW264.7 murine macrophages were cultured in Dulbecco's modified eagle medium (DMEM) containing 10% fetal bovine serum (FBS) as well as 1% U/mL penicillin and streptomycin. Cells were used for further experimentation at 80% confluence.

### Isolation and culture of primary cardiomyocytes

Primary neonatal rat ventricular myocytes (NRVMs) were isolated from neonatal Sprague-Dawley rats (1-2 days old) 52. Briefly, rats were euthanized to collect hearts. After ventricles were minced, myocytes were isolated by serial enzymatic digestion. The isolated cells were plated in culture dishes for 2 h to allow rapid attachment of adherent fibroblasts. Purified cardiomyocytes in the supernatant were collected and cultured for another 48 h before other treatments.

### Observation of cellular uptake by confocal microscopy

H9C2 cells were cultured in 12-well plates at a density of 3 × 10^4^ cells per well in 1 mL of culture medium for 12 h. Then the culture medium was replaced with 1 mL of medium containing 50 μg Cy5-labeled NPs. After incubation for different time periods, cells were washed with PBS, fixed with 4% paraformaldehyde, and stained with 4',6-diamidino-2-phenylindole (DAPI). Subsequently, cells were observed by confocal laser scanning microscopy (CLSM) (Leica, Germany). Similarly, cellular uptake profiles were examined after 2 h of incubation with various doses of Cy5-labeled NPs.

In separate experiments, mitochondrial localization of TPCD NP or TTPCD NP in H9C2 cells was observed by CLSM. In this case, the culture medium was replaced with 1 mL of medium containing 50 μg Cy5-labeled NPs. After incubation for different time periods, cells were stained with MitoTracker Green FM (75 nM) at 37°C for 20 min, washed with PBS, and observed by CLSM.

### Quantification of cellular uptake by flow cytometry

A549 cells, HUVECs, RAW264.7 cells, and H9C2 cells were cultured in 12-well plates at a density of 2 × 10^5^ cells per well in 1 mL of culture medium for 12 h. Then H9C2 cells were treated with fresh medium with or without 2 μM doxorubicin (DOX), while A549 cells, HUVECs, and RAW264.7 cells were incubated without DOX. After 24 h, cells were incubated with 1 mL of medium containing 50 μg Cy5-labeled NPs. Then cells were incubated for different periods of time, followed by flow cytometry analysis (Accuri C6, BD Biosciences). Similarly, cellular uptake was studied after incubation with different doses of Cy5/TPCD NP (varying from 1, 5, 10, 50, to 100 μg/mL) for 2 h.

In another experiments, cellular uptake profiles of TPCD NP or TTPCD NP in H9C2 cells were compared. In this case, H9C2 cells were treated with 2 μM DOX. After 24 h, cells were incubated with 1 mL of medium containing 50 μg Cy5-labeled NPs for different time periods, and then analyzed by flow cytometry analysis.

### ROS generation

H9C2 cells were cultured in dishes at a density of 5 × 10^4^ cells per well in 1 mL of medium for 12 h. After cells were pretreated with different doses of TPCD NP for 2 h, they were stimulated with DOX for 24 h. The control group was treated with medium alone, while the DOX group was treated with DOX without TPCD NP. Subsequently, cells were treated with 2',7'-dichlorofluorescein diacetate (DCFH-DA) in serum-free DMEM. After treatment at 37°C for 20 min, cells were washed with PBS and observed by CLSM. In a separate study, cells were analyzed by flow cytometry following the same procedures. Similarly, flow cytometry analysis of ROS generation was performed in NRVMs.

### Quantification of malondialdehyde (MDA), cardiac troponin I (cTnI), lactate dehydrogenase (LDH), and tumor necrosis factor (TNF)-α

H9C2 cells were cultured in 12-well plates at 1.5 × 10^5^ cells per well in 1 mL of medium for 12 h. Cells were pretreated with different doses of TPCD NP for 2 h and then stimulated with DOX for 24 h. The control group was only treated with medium, while the DOX group was treated with DOX alone. Subsequently, cell culture supernatant was collected and centrifuged at 1000*g* at 4°C for 20 min. The levels of cTnI, LDH, and TNF-α were separately quantified by corresponding kits. Meanwhile, cells were collected and subjected to repeated freeze-thaw processes several times until the cells were fully lysed. Then the supernatant was collected after centrifugation. The level of MDA was detected by a commercial kit following the manufacturer's instructions. Based on similar procedures, biological effects of TTPCD NP and ATTPCD NP were examined in H9C2 cells.

### Animals

Male C57BL/6 (10-12 weeks old) were obtained from the Animal Center of the Third Military Medical University and fed under standard conditions. All animal experiments were performed in line with the Guide for the Care and Use of Laboratory Animals proposed by National Institutes of Health. All the animal care and experimental protocols were performed with review and approval by the Animal Ethical and Experimental Committee of the Third Military Medical University (Army Medical University).

### *In vivo* biodistribution of inhaled TPCD NP

Male C57BL/6 mice were exposed to an inhalation chamber nebulized with Cy7.5-labeled NPs at a theoretical dose of 50 mg/kg in 5 mL of saline. At different time points after inhalation, the heart and other major organs were collected. *Ex vivo* imaging was performed by an IVIS Spectrum living imaging system.

To estimate the exact dose after inhalation delivery, intratracheal nebulization and inhalation were compared. In this case, mice were divided into three groups (n = 3 per group), *i.e.*, the control, intratracheal nebulization, and inhalation groups. Mice in the intratracheal nebulization group were treated by inhalation via intratracheal nebulization of Cy7.5-labeled NPs (25 mg/kg). Mice in the inhalation group were exposed to the inhalation chamber nebulized with Cy7.5-labeled NPs (25 mg/kg) in 5 mL of saline. The same volume of saline was given to mice in the control group. Immediately after different treatments, lungs were collected for *ex vivo* imaging. In all cases, fluorescence intensities were calculated by the Living Image software.

### Intrapulmonary distribution of Cy5/TPCD NP

Mice were treated with Cy5/TPCD NP at a theoretical dose of 50 mg/kg by inhalation. After 24 h, lungs were collected, fixed with 4% paraformaldehyde, and embedded in paraffin. Then lung sections were prepared and incubated with antibodies to EpCAM (1:100), CD31 (1:500), and CD68 (1:200) for 24 h, followed by incubation with the secondary antibody Alexa Fluor 488-labeled goat anti-rabbit IgG (H+L). After staining with DAPI, lung sections were visualized by CLSM. In addition, immunofluorescence analysis was performed for heart tissues collected at 60 h after inhalation.

In separate experiments, the cellular distribution of Cy5/TPCD NP in pulmonary tissues was analyzed by flow cytometry. Briefly, mice were nebulized with Cy5/TPCD NP at a theoretical dose of 50 mg/kg. Control mice were nebulized with saline. After 24 h, mice were sacrificed to isolate lungs. Then lungs were minced and incubated in 5 mL of DMEM containing 1.3 U Liberase TM and 1.25 mg DNase I at 37°C for 30 min, followed by filtering with a 40 µm cell strainer and incubation with red blood cell lysis buffer for 1 min. The obtained suspension was centrifuged at 400*g*, and cells were resuspended in PBS containing 0.5% bovine serum albumin. After suspended cells (1 × 10^6^) were stained with antibodies to CD45R, CD326, CD31, F4/80, and CD11b, flow cytometry analysis was performed.

### Distribution of inhaled TPCD NP in blood cells

In a separate study, the distribution of TPCD NP in different cells was analyzed by flow cytometry using the previously established method 53. Briefly, mice were nebulized with Cy5/TPCD NP at a theoretical dose of 50 mg/kg. At 12 h after inhalation, whole blood samples were collected. Then cell suspensions were stained with different antibodies 54, followed by flow cytometry analysis.

### Prevention of DOX-induced heart failure by different nanotherapies in mice

Mice were divided into five groups (n = 5 per group), including the control, DOX, DOX+TPCD NP (4 mg/kg), DOX+TPCD NP (10 mg/kg), and DOX+TPCD NP (25 mg/kg) groups. Mice in the DOX and DOX+TPCD NP groups were subjected to a single intraperitoneal injection of DOX (15 mg/kg). An equal volume of saline was given to mice in the control group. Beginning from 2 days before DOX injection, mice in the DOX+TPCD NP groups were nebulized with TPCD NP (at 4, 10, or 25 mg/kg) every day for 7 days. Mice in the control and DOX groups were nebulized with an equal volume of saline. At day 7 after different treatments, mice were euthanized. The hearts, tibias, and other major organs (including the liver, spleen, lung, and kidney) were harvested. The organ index was calculated as the ratio of the organ weight to the tibia length. Blood samples were collected for blood routine and biochemical analyses.

To compare *in vivo* efficacies of dexrazoxane (DEX) and TPCD NP, mice in the DEX group were treated by a single intraperitoneal injection of DEX (200 mg/kg) at 2 h before administration of DOX. For the TPCD NP group, mice were nebulized with TPCD NP (25 mg/kg) 2 days before administration of DOX. Then similar procedures were followed as described above.

In a separate study, mice were nebulized with TPCD NP or TTPCD NP at 25 mg/kg 2 days before administration of DOX. After 7 days, hearts and tibias were harvested to calculate the ratio of the heart weight to the tibia length. Through similar procedures, therapeutic effects of ATTPCD NP were evaluated. In this case, the dose of ATTPCD NP was 25 mg/kg, while Ac2-26 at 14.75 µg/kg and the same dose of TTPCD NP served as the controls.

### Echocardiography

Mice were anesthetized with 1-1.5% isoflurane. Cardiac function was determined by echocardiography (VisualSonics Vevo 2100 Imaging System). Left ventricular ejection fraction (LVEF) and left ventricular fractional shortening (LVFS) were measured from M-mode images obtained from the parasternal short-axis view at the papillary muscle level.

### Measurements of serum levels of cTnI, creatine kinase-MB isoenzyme (CK-MB), and creatine kinase (CK)

Blood samples were collected and allowed to stand at room temperature for 2 h before centrifugation (1000*g*) at 4°C for 20 min. The levels of cTnI, CK-MB, and CK were measured by ELISA assay.

### Evaluation of oxidative stress and quantification of inflammatory cytokines

Dihydroethidium (DHE) staining was used to detect superoxide anion generation. To this end, fresh heart slices were incubated with DHE (1:200) at 37°C in dark for 30 min. Images were acquired by fluorescence microscopy (Olympus, Japan). Image-pro Plus 6.0 software was used to analyze fluorescence intensities.

To measure the levels of MDA, H_2_O_2_, TNF-α, and myeloperoxidase (MPO) in the myocardium, heart tissues were isolated and homogenized in PBS on ice. The supernatant was collected after centrifugation at 5000*g* for 5 min, followed by ELISA using corresponding kits.

### Acute toxicity evaluation of TPCD NP in mice

Mice were randomly divided into three groups (n = 5 per group), including the control, TPCD NP at 62.5 mg/kg (*i.e.*, TPCD NP-L), and TPCD NP at 125 mg/kg (*i.e.*, TPCD NP-H) groups. Mice in the TPCD NP-L and TPCD NP-H groups were nebulized with TPCD NP at 62.5 or 125 mg/kg every day for 7 days, respectively. Mice in the control group were nebulized with an equal volume of saline. The body weight of mice was recorded. After 7 days, mice were euthanized. Blood samples were collected for route blood tests and biochemical analyses. The main organs were isolated and the organ index was calculated as the ratio of organ weight to body weight. The wet/dry weight ratio of lung tissues was determined to evaluate the severity of pulmonary edema. To this end, lungs were first weighed to get the wet weight, and then the lung tissues were dried at 60°C for 48 h to get dry weight. Meanwhile, the histological sections were stained with hematoxylin and eosin (H&E). In a separate study, bronchoalveolar lavage fluid was collected. After centrifugation at 16000*g* for 10 min, the supernatant was collected for quantification of the levels of TNF-α and interleukin (IL)-1β by ELISA.

### Statistical analysis

Quantitative data are expressed as mean ± standard deviation (SD). Statistical analyses were performed by SPSS 20.0 using the one-way ANOVA test for experiments with three or more groups, while the unpaired t-test was used for data with two groups. Statistical significance was considered at *P* < 0.05.

## Results

### Preparation and characterization of a bioactive nanoparticle-derived nanotherapy

According to our previously established method 49, an antioxidative and anti-inflammatory material (defined as TPCD) was synthesized by conjugating Tempol and a phenylboronic acid pinacol ester (PBAP) unit onto β-cyclodextrin (β-CD), a cyclic oligosaccharide with excellent *in vivo* safety 55. The structure of synthesized TPCD was confirmed by Fourier transform infrared (FT-IR) and ^1^H NMR spectroscopy (Figure S1A-B). Calculation according to proton signals in the ^1^H NMR spectrum of TPCD suggested that approximately 2 Tempol and 5 PBAP were conjugated onto each β-CD molecule.

TPCD nanoparticles (defined as TPCD NP) were produced by a modified nanoprecipitation/self-assembly method 49, in the existence of lecithin and an amphiphilic conjugate DSPE-PEG (Figure 1B). Thus obtained NPs generally display core-shell structure, with a hydrophobic core coated by a lecithin shell, in which DSPE-PEG chains are anchored via DSPE moieties 51, 56. The peripherally distributed PEG chains can provide resulting NPs with good colloidal stability and ensure their desirable penetration capability in mucus 38, 57. Observation by transmission electron microscopy (TEM) and scanning electron microscopy (SEM) revealed a well-defined spherical shape of TPCD NP (Figure 1C-D). Measurement by dynamic light scattering indicated that TPCD NP showed a narrow size distribution (Figure 1E), with the average diameter of 101 nm and ζ-potential of -29.4 ± 1.7 mV.

We then tested *in vitro* ROS-scavenging ability of TPCD NP. With an increase in the TPCD NP concentration, the amount of scavenged H_2_O_2_ notably increased, showing a nearly linear profile (Figure 1F). Similarly, TPCD NP effectively eliminated superoxide anion in a dose-dependent manner (Figure 1G). In addition, both time- and dose-dependent DPPH free radical scavenging profiles were observed (Figure 1H).

### *In vitro* biological activities of TPCD NP

**Figure 2:**
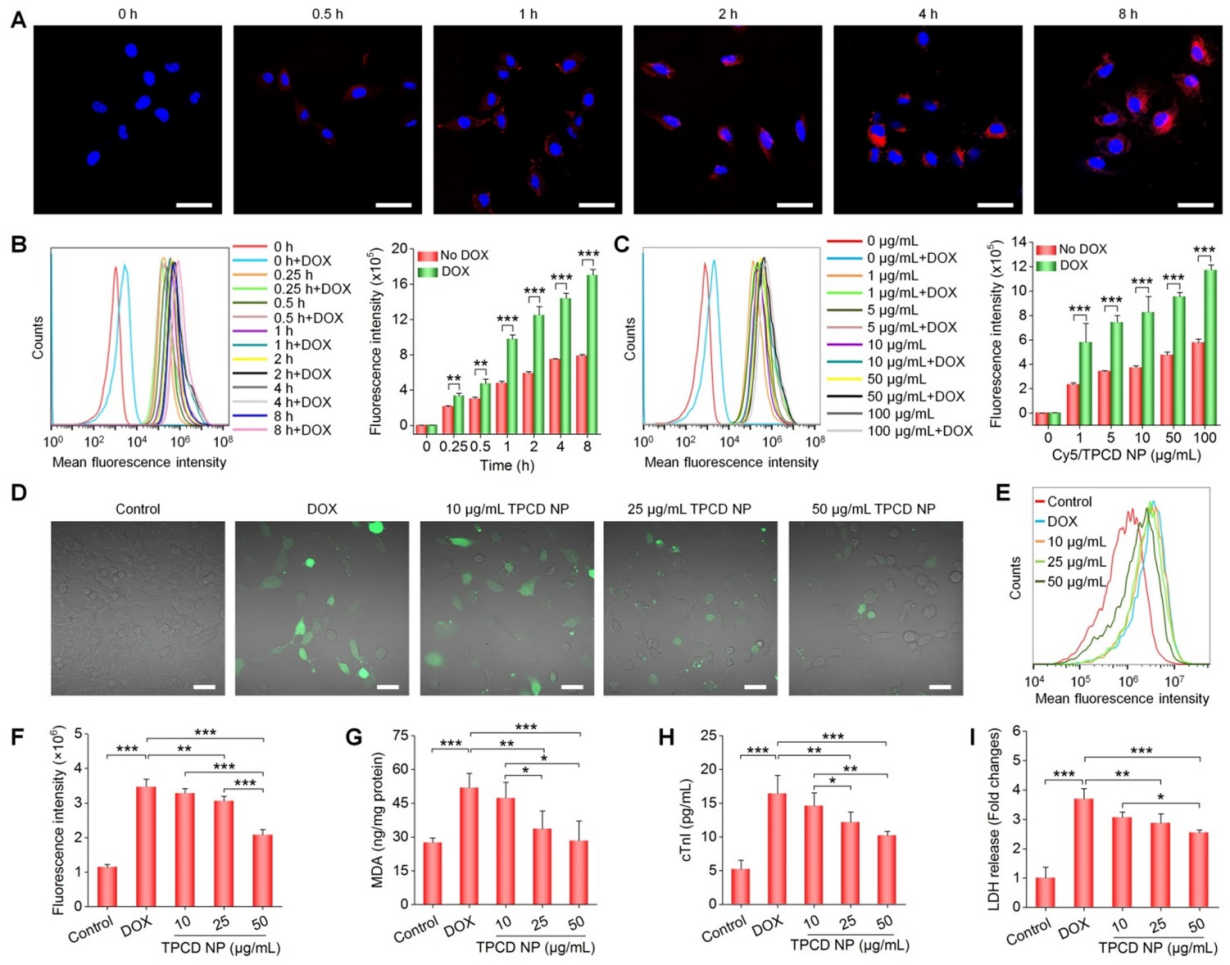
Cellular uptake of TPCD NP and *in vitro* biological effects in H9C2 cells. (A) Confocal microscopy images showing time-dependent cellular uptake of Cy5/TPCD NP. (B-C) Flow cytometric curves (left) and quantitative analysis (right) of time-dependent (B) or dose-dependent (C) cellular uptake of Cy5/TPCD NP in H9C2 cells with or without DOX treatment. For time-dependent experiments, the dose of Cy5/TPCD NP was 50 µg/mL, while the incubation time was 2 h for dose-response studies. (D-F) Representative fluorescence images (D) and flow cytometric quantification (E-F) of intracellular ROS generation after stimulation with DOX and treatment with different doses of TPCD NP. (G) Intracellular MDA levels after different treatments. The protein content was measured by the BCA assay. (H-I) Expression levels of cTnI (H) and LDH (I). Scale bars: 40 μm (A, D). Data are mean ± SD (B, C, F, n = 3; G-I, n = 4). Statistical significance was assessed by the unpaired t-test for data in (B-C) and the one-way ANOVA with post-hoc LSD tests for data in (F-I). *P < 0.05, **P < 0.01, ***P < 0.001.

We first examined cellular uptake of TPCD NP in H9C2 rat cardiomyoblast cells, using Cy5-labeled TPCD NP (Cy5/TPCD NP). Confocal microscopy observation indicated efficient internalization of Cy5/TPCD NP by H9C2 cells in a time-dependent manner (Figure 2A). Significant intracellular fluorescent signals were observed even at 0.5 h after incubation. In addition, a clearly dose-response cellular uptake profile was found in H9C2 cells after 2 h of incubation (Figure S2). Further flow cytometry analysis affirmed both time- and dose-dependent cellular internalization of Cy5/TPCD NP in H9C2 cells (Figure 2B-C). Consistently, we found that Cy5/TPCD NP could be efficiently endocytosed by neonatal rat ventricular myocytes (NRVMs) (Figure S3). Moreover, cellular uptake efficiency of Cy5/TPCD NP in DOX-stimulated H9C2 cells was significantly higher than that of cells without DOX pretreatment, regardless of incubation time periods (Figure 2B). Also, DOX-treated H9C2 cells exhibited significantly higher cellular internalization at various doses of Cy5/TPCD NP, as compared to those without DOX pretreatment (Figure 2C). Since TPCD NP will be delivered via inhalation, we also examined cellular uptake profiles in other cells related to the absorption and translocation of inhaled TPCD NP, including A549 human lung epithelial cells, HUVECs, and RAW264.7 murine macrophages. All these tested cells showed effective endocytosis of Cy5/TPCD NP in time- and dose-dependent manners (Figure S4-6).

ROS-induced oxidative stress in cardiomyocytes plays an important role in the pathogenesis of heart failure 58, 59. Therefore, we evaluated *in vitro* anti-oxidative effects of TPCD NP in H9C2 cells. Direct observation via confocal microscopy revealed a notably enhanced ROS level in H9C2 cells treated with DOX (Figure 2D). This is consistent with the previous finding that DOX can cause abnormally increased intracellular ROS in cardiomyocytes 60. Pretreatment with TPCD NP notably reduced ROS levels in DOX-stimulated H9C2 cells. The dose-dependent anti-oxidative effect of TPCD NP in DOX-induced H9C2 cardiomyocytes was further confirmed by flow cytometric quantification (Figure 2E-F). In line with significantly reduced oxidative stress in H9C2 cardiomyocytes by TPCD NP, treatment with this nanotherapy also effectively inhibited the production of MDA (Figure 2G), which is one of the end products of lipid peroxidation 48. Moreover, DOX-treated H9C2 cells expressed significantly higher levels of cardiac troponin I (cTnI) and lactate dehydrogenase (LDH) (Figure 2H-I), two typical markers of myocardial injury 61. Pretreatment with TPCD NP effectively reduced expressions of cTnI and LDH by H9C2 cells. Also, anti-oxidative and protective effects of TPCD NP were detected in NRVMs (Figure S7). Collectively, these results demonstrated that TPCD NP can protect cardiomyocytes from DOX-induced oxidative stress and cell damage.

### Heart targeting capability and mechanistic studies of inhaled TPCD NP

**Figure 3:**
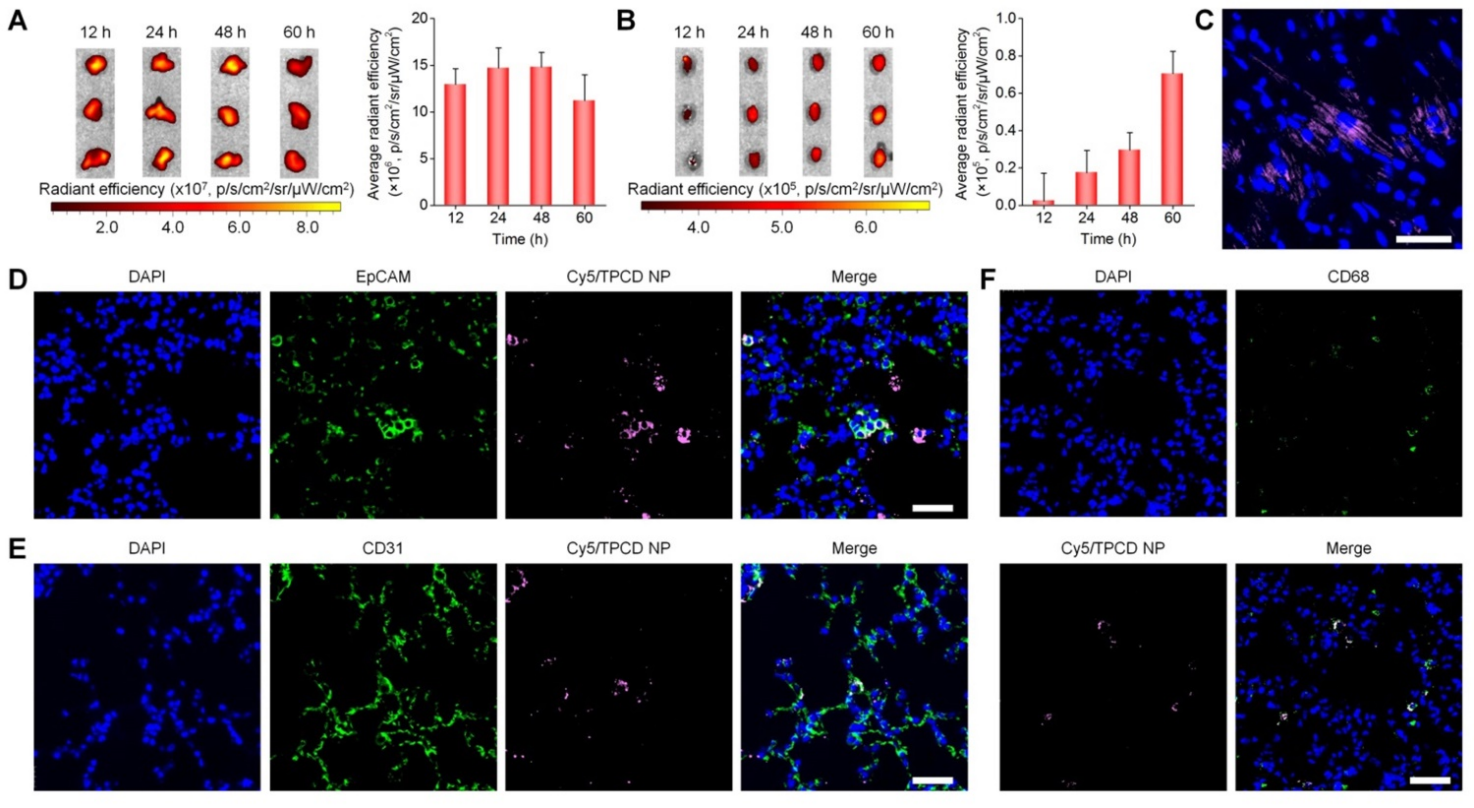
Distribution of TPCD NP in the lungs and hearts after inhalation in mice. (A-B) *Ex vivo* fluorescent images (left) and quantitative analysis (right) of mouse lungs (A) and hearts (B) at various time points after inhalation of Cy7.5/TPCD NP. (C) A typical fluorescence image shows the distribution of Cy5/TPCD NP in a cryosection of the heart at 60 h after inhalation. (D-F) Immunofluorescence analysis of co-localization of Cy5/TPCD NP with EpCAM^+^ lung epithelial cells (D), CD31^+^ lung endothelial cells (E), and CD68^+^ macrophages (F) in lung sections. Scale bars: 40 μm. Data in (A-B) are mean ± SD (n = 3).

To assess whether TPCD NP can be delivered to the heart after inhalation, *in vivo* distribution profiles of inhaled TPCD NP in typical major organs were first examined in mice. At 2 h after inhalation of Cy7.5/TPCD NP, considerable accumulation of Cy7.5 fluorescence was clearly observed in the lungs (Figure S8A), showing a notable increase at detected time points from 2 to 12 h. Nevertheless, evident fluorescence in the heart was only found at 12 h after inhalation of Cy7.5/TPCD NP (Figure S8B). In a separate study, we found slightly increased Cy7.5 fluorescence intensities in lungs when detection was prolonged from 12 to 48 h (Figure 3A), followed by a decrease at 60 h after Cy7.5/TPCD NP inhalation. This suggested that pulmonary deposition of TPCD NP was largely completed within 12 h. Correspondingly, fluorescence intensities in the heart significantly increased when time varied from 12 to 60 h after inhalation (Figure 3B). The heart accumulation of Cy5/TPCD NP was further affirmed by confocal microscopic observation of the heart cryosection (Figure 3C). As for other organs, Cy7.5/TPCD NP displayed similar time-dependent distribution profiles in the liver and spleen (Figure S9), while the Cy7.5/TPCD NP accumulation in kidneys resembled that of the heart. Notably, whereas the lung accumulation of Cy7.5/TPCD NP considerably decreased at 60 h after inhalation, the heart distribution still increased at the examined time points from 60 to 120 h (Figure S10). These results indicated that inhaled Cy7.5/TPCD NP can be gradually absorbed and translocated to other major organs.

**Figure 4:**
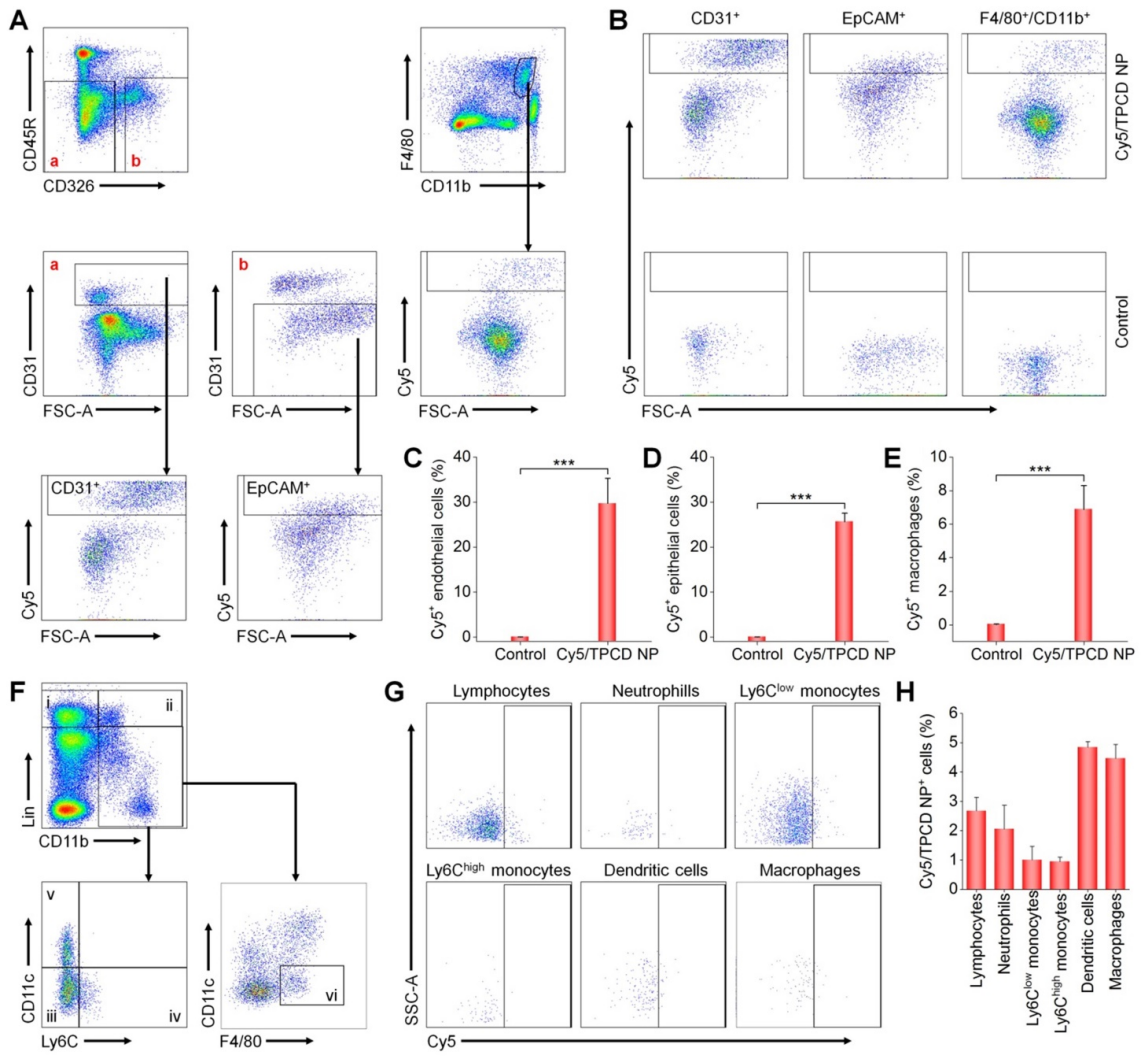
Flow cytometric analysis of the distribution of Cy5/TPCD NP in different pulmonary cells and blood cells. (A) The gating strategy used for flow cytometry analysis of lung cells at 24 h after inhalation of Cy5/TPCD NP. Biomarkers for lung epithelial cells (EpCAM^+^), lung endothelial cells (CD31^+^), and macrophages (F4/80^+^ and CD11b^+^) were separately used to distinguish different sub-types. (B) Typical dot plots show Cy5 distribution in control and Cy5/TPCD NP-treated mouse lung tissues. (C-E) Quantified Cy5^+^ cell proportions in pulmonary endothelial cells (C), epithelial cells (D), and macrophages (E). (F) The gating strategy used for flow cytometry analysis of blood cells at 12 h after inhalation of Cy5/TPCD NP. i, lymphocytes; ii, neutrophils; iii, Ly6C^low^ monocytes; iv, Ly6C^high^ monocytes; v, dendritic cells; and vi, macrophages. (G) Typical dot plots show Cy5 distribution in different blood cells. (H) Quantified Cy5^+^ cell proportions in lymphocytes, neutrophils, Ly6C^low^ monocytes, Ly6C^high^ monocytes, dendritic cells, and macrophages. Data in (C-E,H) are mean ± SD (n = 4). Statistical significance was assessed by the unpaired t-test. ***P < 0.001.

Whereas our results and other studies on different nanomaterials revealed the transport of NPs from the lungs to the systemic circulation, the underlying mechanisms dominating intrapulmonary translocation of NPs remain unknown 26, 62. Therefore we further explored the transport pathways responsible for heart targeting of inhaled Cy5/TPCD NP. Immunofluorescence analysis revealed the co-localization of Cy5/TPCD NP with EpCAM^+^ epithelial cells, CD31^+^ endothelial cells, and CD68^+^ macrophages in lungs after inhalation (Figure 3D-F). The distribution of Cy5/TPCD NP in different cells of the lung tissue was further quantified by flow cytometry (Figure 4A-B). It was found that 29.7±5.6% lung endothelial cells were Cy5-positive (Figure 4C), while 25.6±1.9% epithelial cells contained Cy5/TPCD NP (Figure 4D). By contrast, only a very low proportion of lung macrophages (6.9±1.4%) were Cy5-positive (Figure 4E), which should be resulting from the relatively low macrophage count in the lungs. Consequently, absorption and translocation of Cy5/TPCD NP were mainly mediated by epithelial and endothelial cells.

Since previous studies reported that inhaled NPs can be transported into the blood circulation through the pulmonary lymphatic system 63, we also examined the distribution of Cy5/TPCD NP in different peripheral blood cells by flow cytometry (Figure 4F-H). Although we found the localization of Cy5/TPCD NP in different blood cells including lymphocytes, neutrophils, Ly6C^low^ monocytes, Ly6C^high^ monocytes, dendritic cells, and macrophages, the proportion of Cy5-positive cells for each cell type was less than 5%. This can be explained by the fact that only a small amount of Cy5/TPCD NP was transported to the blood circulation. Accordingly, lymphatic transport was not the main route accounting for the heart accumulation of Cy5/TPCD NP.

Collectively, our results suggested that inhaled TPCD NP will first deposit in the lungs, followed by translocation across the pulmonary epithelium and endothelium, which can be mediated by lung epithelial cells and endothelial cells, respectively, via transcellular and/or paracellular pathways. TPCD NP thus delivered to pulmonary capillaries will be further transported to the heart via the pulmonary circulation, since this is the shortest route for transportation between the heart and lungs, in which TPCD NP might be transported from the pulmonary veins to the left atrium and then deposited in the heart via the coronary arteries. In addition to accumulation in the heart, a considerable proportion of TPCD NP will be transported by the blood pumped out of the heart to other organs via the systemic circulation.

### Prevention of DOX-induced heart failure by inhaled TPCD NP in mice

**Figure 5:**
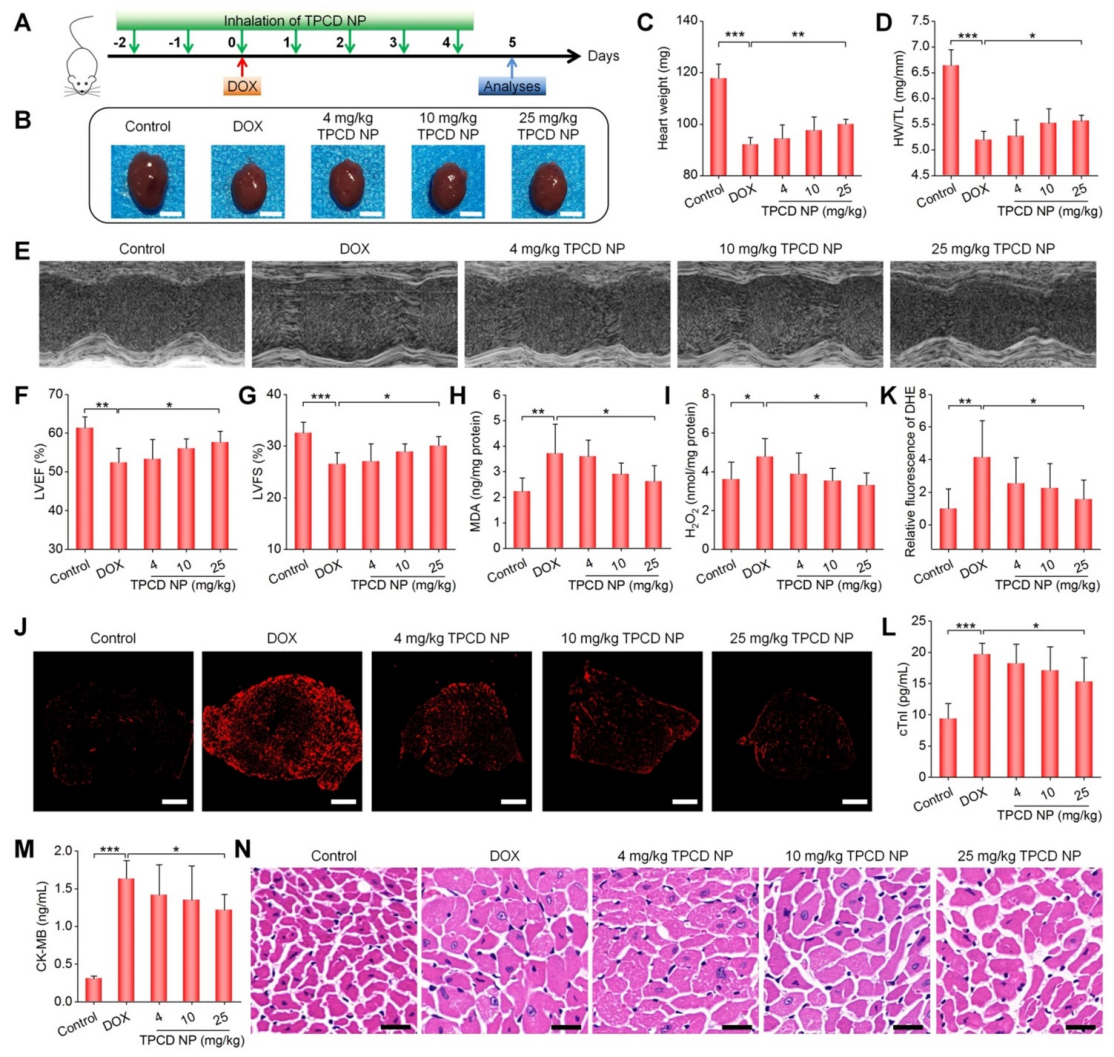
Therapeutic effects of inhaled TPCD NP on DOX-induced heart failure in mice. (A) Schematic illustration of the treatment protocols. (B) Typical digital photos show hearts isolated from mice in different groups. (C) The heart weight of different groups. (D) Ratios of HW/TL for different groups. (E) Representative M-mode echocardiography images of mouse hearts after different treatments. (F-G) Left ventricular ejection fraction (LVEF) and left ventricular fraction shortening (LVFS) quantified by echocardiography. (H-I) The levels of MDA (H) and H_2_O_2_ (I) in tissue homogenates of hearts from mice treated with different formulations. The total protein content in heart homogenates was measured by the BCA assay. (J-K) Fluorescence images (J) and quantitative analysis (K) of DHE-stained heart cryosections for mice subjected to different treatments. (L-M) Serum levels of cTnI (L) and CK-MB (M). (N) H&E-stained histological sections of hearts. Control, healthy mice treated with saline; DOX, mice treated with DOX and saline. In different TPCD NP groups, diseased mice were treated with different doses of TPCD NP. Scale bars: 3 mm (B), 500 μm (J), 20 μm (N). Data in (C-D,F-I,K-M) are mean ± SD (n = 5). Statistical significance was assessed by the one-way ANOVA with post-hoc LSD tests. *P < 0.05, **P < 0.01, ***P < 0.001.

Based on the above results, we examined *in vivo* efficacies of TPCD NP. A mouse model of heart failure was induced by intraperitoneal injection of DOX, since this approach has been widely reported 64-66. Of note, DOX-induced toxic models of cardiomyopathy are highly specific forms of injury, which are also useful in assessing cardiac responses to oxidative stress 67. In TPCD NP groups, mice were pre-treated with TPCD NP by inhalation at varied theoretical doses (Figure 5A), considering gradual accumulation of inhaled TPCD NP in the heart. For the model group treated with DOX alone, we found a significant decrease in the heart weight and the ratio of heart weight to tibia length (HW/TL) (Figure 5B-D). Treatment with the nanotherapy TPCD NP notably increased both heart weight and HW/TL, in a dose-response pattern. Cardiac function of mice was further evaluated by echocardiography. Representative M-mode echocardiographic images showed that DOX significantly reduced the waveform range and weakened the wall motion (Figure 5E). Quantitative analysis revealed the significantly reduced left ventricle ejection fraction (LVEF) and left ventricle fraction shortening (LVFS) in the DOX group (Figure 5F-G), compared to those of the control healthy group. By contrast, treatment with TPCD NP remarkably alleviated cardiac dysfunction as well as reduced LVEF and LVFS caused by DOX.

Since oxidative damage is the main cause of myocardial injury 58, 59, 68, 69, we further examined the degree of cardiac oxidative stress in heart tissues after different treatments. As compared to the control group, the DOX group exhibited significantly high levels of MDA and H_2_O_2_ in the myocardium (Figure 5H-I). Moreover, fluorescence observation of heart cryosections stained with dihydroethidium (DHE, a fluorescent probe for superoxide anion) revealed the strongest fluorescence in the model group, while therapy with TPCD NP notably reduced fluorescence intensities (Figure 5J-K). Further quantification of serum levels of cTnI and CK-MB, two typical biomarkers of cardiac injury, also substantiated that TPCD NP treatment at 25 mg/kg effectively inhibited DOX-induced myocardial injury (Figure 5L-M). In addition, inspection on H&E-stained heart sections showed notable cytoplasmic vacuolization and nuclear swelling of myocardial cells in the model group (Figure 5N), while these pathological abnormalities were considerably reduced in TPCD NP-treated mice, particularly at 25 mg/kg. Moreover, treatment with TPCD NP could also reverse DOX-induced reduction in the ratios of organ weight to tibia length for other major organs (Figure S11). Likewise, TPCD NP therapy enhanced the number of white blood cells and platelets as well as reduced serum levels of alanine aminotransferase (ALT) and aspartate aminotransferase (AST) (Figure S12). Notably, the lung accumulation efficiency of inhaled Cy7.5/TPCD NP was estimated by comparing fluorescence intensities with those of Cy7.5/TPCD NP administered by direct intratracheal delivery (Figure S13). The result indicated that approximately 4% TPCD NP was deposited in the lungs after inhalation. Consequently, the theoretical inhalation doses of 4, 10, and 25 mg/kg TPCD NP are actually 0.16, 0.4, and 1 mg/kg TPCD NP, respectively.

Taken together, all these results demonstrated that treatment with the nanotherapy TPCD NP by inhalation can efficaciously ameliorate DOX-induced heart failure in mice at a very low actual dose of 1 mg/kg, largely by site-specific attenuation of oxidative stress in the heart. To a certain degree, DOX-mediated side effects on other organs or tissues can also be mitigated by inhaled TPCD NP.

### Comparison of *in vivo* efficacy of TPCD NP with dexrazoxane in mice with DOX-induced heart failure

**Figure 6:**
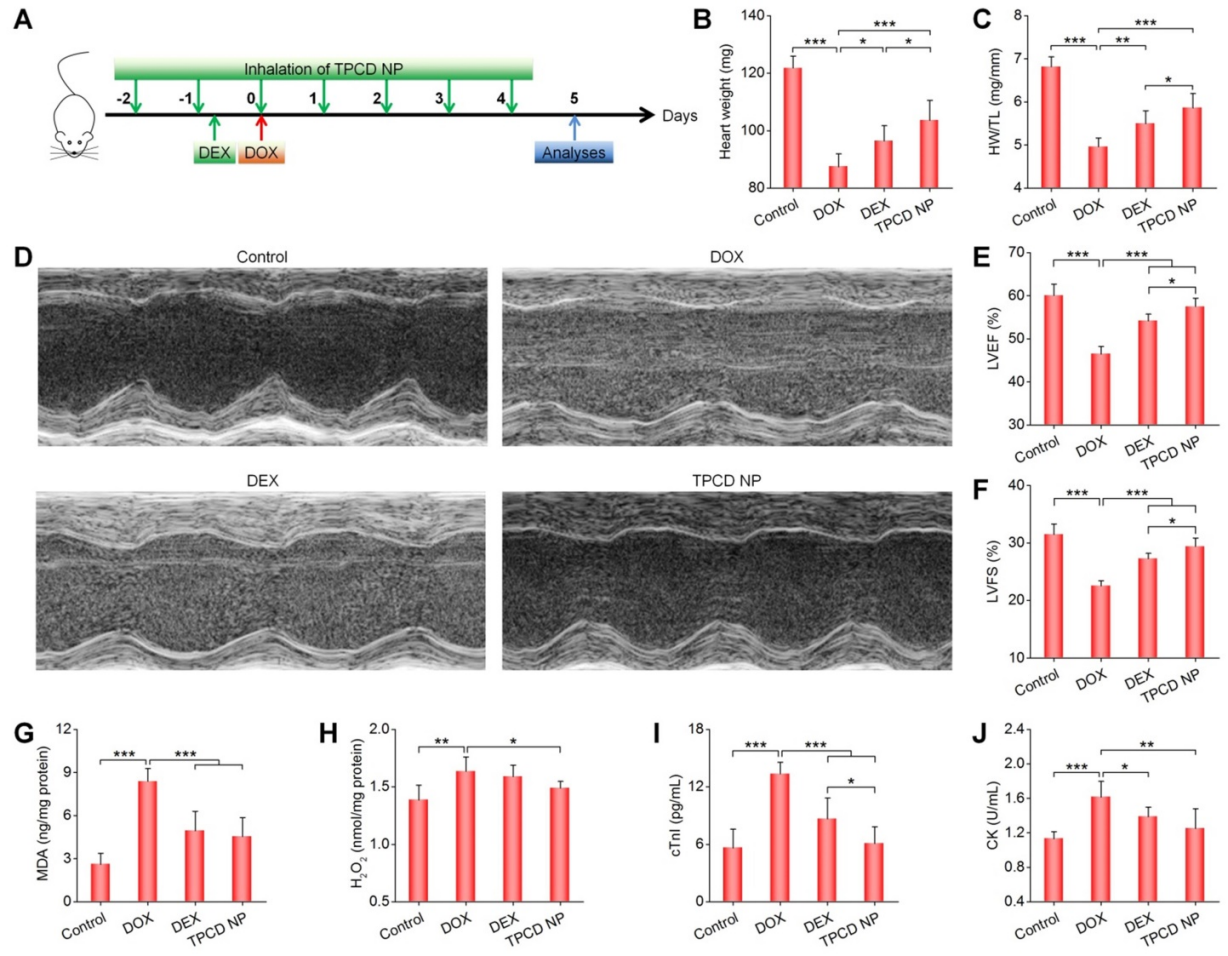
Comparison of therapeutic effects of dexrazoxane (DEX) and TPCD NP in mice with DOX-induced heart failure. (A) Schematic illustration of the treatment protocols. (B) The heart weight of different groups. (C) Ratios of HW/TL for different groups. (D) Representative M-mode echocardiography images of mouse hearts after different treatments. (E-F) LVEF and LVFS quantified by echocardiography. (G-H) The levels of MDA (G) and H_2_O_2_ (H) in tissue homogenates of hearts isolated from mice treated with different formulations. The total protein content in heart homogenates was measured by the BCA assay. (I-J) Serum levels of cTnI (I) and CK (J). Control, healthy mice treated with saline; DOX, mice treated with DOX and saline; DEX, mice treated with DOX and DEX at 200 mg/kg; TPCD NP, mice treated with DOX and TPCD NP at 25 mg/kg. Data in (B-C,E-J) are mean ± SD (n = 5). Statistical significance was assessed by the one-way ANOVA with post-hoc LSD tests. *P < 0.05, **P < 0.01, ***P < 0.001.

Subsequently, we compared *in vivo* therapeutic effects of inhaled TPCD NP with dexrazoxane (DEX), a cardiac protective drug approved by FDA for DOX-induced dysfunction 70. For the DEX group, mice were intraperitoneally injected with DEX (200 mg/kg) at 2 h before administration of DOX, while the TPCD NP group was treated by inhalation at 25 mg/kg (Figure 6A). Whereas treatment with either DEX or TPCD NP significantly increased the heart weight and HW/TL ratio as well as remarkably improved cardiac dysfunction by increasing the DOX-induced abnormal reduction in LVEF and LVFS (Figure 6B-F), significant differences were found between the TPCD NP and DEX groups. Correspondingly, the levels of MDA and H_2_O_2_ in the myocardium as well as serum levels of cTnI and CK were considerably decreased after treatment with DEX or TPCD NP (Figure 6G-J), and more beneficial outcomes were achieved by TPCD NP. These results substantiated that inhaled TPCD NP at 25 mg/kg was more effective than the currently used cardiac protective drug DEX at a clinically relevant dose for the treatment of DOX-induced heart failure in mice.

### Engineering of a TPCD NP-derived mitochondrial-targeting nanotherapy for heart failure

Mitochondria, which are abundant in the heart, are the key sites of ROS generation in myocardial dysfunction induced by DOX 71. In addition, previous studies demonstrated that mitochondrial-targeting antioxidants are more effective than those of non-targeting ones in the treatment of various diseases related to mitochondrial-mediated oxidative stress 72-74. Consequently, functionalization of nanotherapies with mitochondrial-targeting moieties is a promising strategy for simultaneously enhancing their heart targeting capability and anti-oxidative activity.

**Figure 7:**
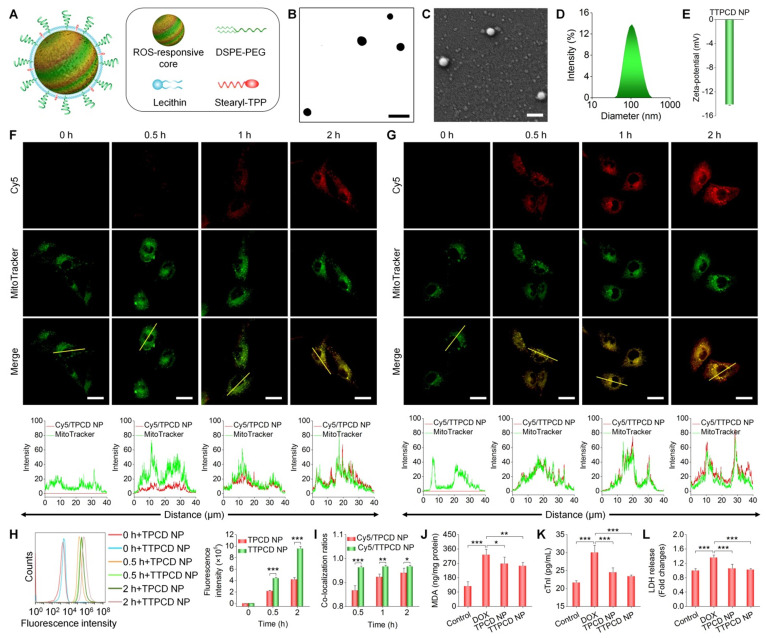
Design, engineering, and biological effects of a mitochondrial-targeting nanotherapy TTPCD NP. (A) Schematic of TPP-decorated TPCD NP (TTPCD NP). (B-E) TEM (B) and SEM (C) images as well as size distribution (D) and zeta-potential (E) of TTPCD NP. (F-G) Confocal microscopy images showing the mitochondrial localization of Cy5/TPCD NP (F) and Cy5/TTPCD NP (G) in H9C2 cells. Images in the lower panels show quantitative analysis of fluorescence intensities along the yellow lines on single cells in indicated images. (H) Flow cytometric curves (left) and quantitative analysis (right) of time-dependent cellular uptake of Cy5/TPCD NP and Cy5/TTPCD NP at 50 µg/mL in H9C2 cells stimulated with DOX. (I) The co-localization ratios of Cy5/TPCD NP or Cy5/TTPCD NP with mitochondria. (J) Intracellular MDA levels after DOX stimulation and treatment with TPCD NP or TTPCD NP at 50 µg/mL. The protein content was measured by the BCA assay. (K-L) Expression levels of cTnI (K) and LDH (L) by H9C2 cells after stimulation with DOX and treatment with TPCD NP or TTPCD NP. Scale bars: 200 nm (B-C), 20 μm (F-G). Data are mean ± SD (E,H-I, n = 3; J-L, n = 4). Statistical significance was assessed by the unpaired t-test for data in (H-I) and the one-way ANOVA with post-hoc LSD tests for data in (J-L). *P < 0.05, **P < 0.01, ***P < 0.001.

As a proof of concept, herein TPCD NP was functionalized with STPP (Figure S14), since previous studies demonstrated its mitochondrial-targeting capacity 50, 75. STPP-decorated TPCD NP (defined as TTPCD NP) was also formulated by the aforementioned self-assembly/nanoprecipitation method, using TPCD, lecithin, DSPE-PEG, and STPP (Figure 7A). TTPCD NP displayed a spherical shape (Figure 7B-C), with the mean diameter of 104 ± 3 nm and ζ-potential of -14.3 mV (Figures 7D-E). Similar to TPCD NP, Cy5-labeled TTPCD NP (Cy5/TTPCD NP) also showed time-dependent cellular uptake in H9C2 cells (Figure 7F-G). Nevertheless, flow cytometric quantification indicated that TTPCD NP was more effectively internalized by H9C2 cardiomyocytes (Figure 7H). Further, mitochondrial-targeting capability of both nanotherapies was examined after staining with a fluorescent dye MitoTracker (green). Although both Cy5/TPCD NP and Cy5/TTPCD NP showed mitochondrial-targeting capacity (Figure 7F-G), quantitative analysis revealed that the co-localization ratios of Cy5/TTPCD NP were significantly higher than those of Cy5/TPCD NP at the examined time points (Figure 7I). These results demonstrated that both cellular uptake and mitochondrial-targeting capability of TPCD NP can be significantly enhanced after decoration with STPP, thereby affording a more efficient nanotherapy TTPCD NP. In line with this finding, TTPCD NP more effectively protected H9C2 cells from DOX-induced oxidative stress injury, compared to TPCD NP, as implicated by notably decreased MDA, cTnI, and LDH (Figure 7J-L). Collectively, these findings substantiated that TTPCD NP afforded additionally enhanced therapeutic effects, by increasing endocytosis and mitochondrial accumulation.

### *In vivo* heart targeting and cardiacprotective effects of TTPCD NP in mice

**Figure 8:**
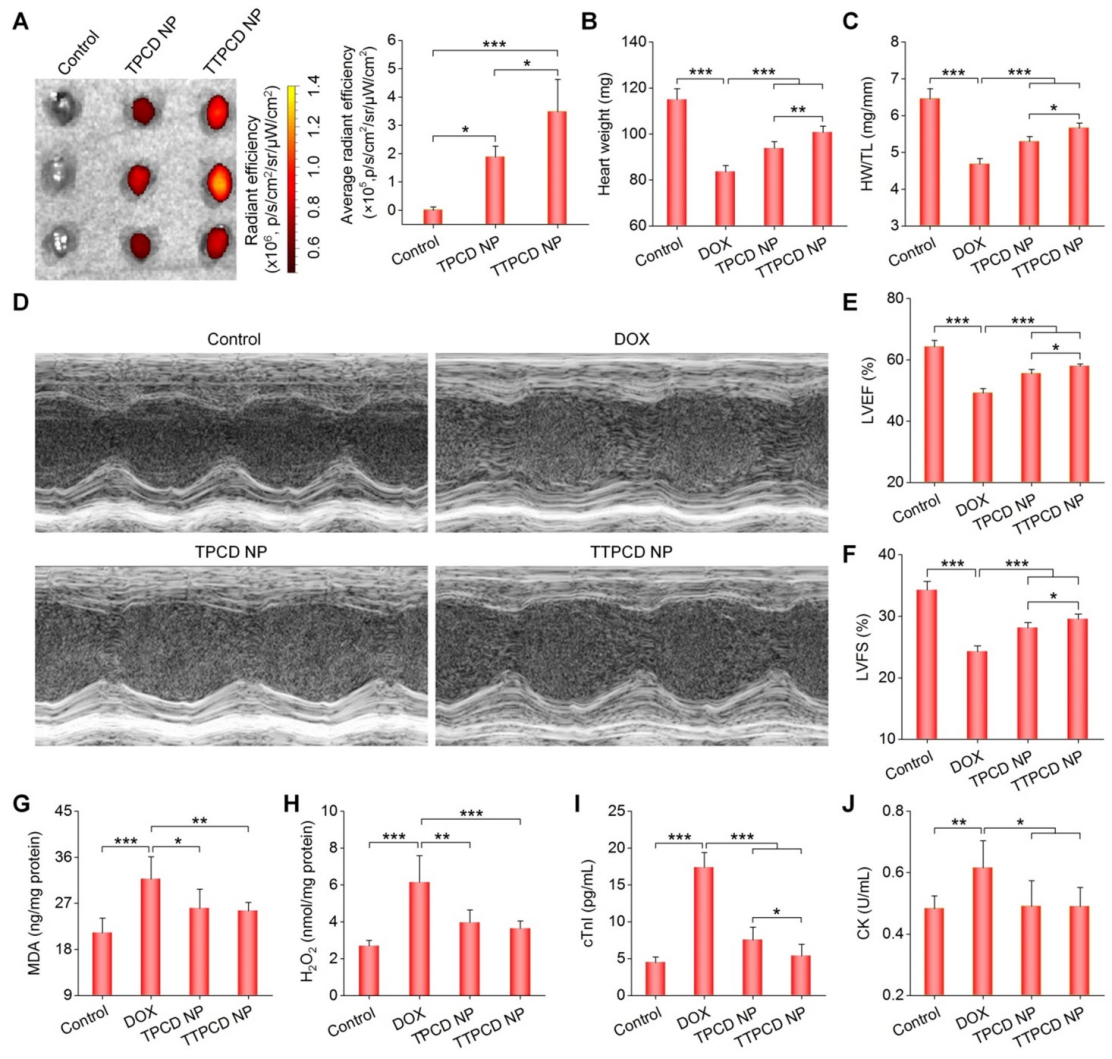
*In vivo* heart targeting and therapeutic effects of a mitochondrial-targeting nanotherapy TTPCD NP in mice. (A) *Ex vivo* fluorescence images (left) and quantitative analysis (right) of hearts isolated from mice after inhalation of Cy7.5/TPCD NP or Cy7.5/TTPCD NP. (B) The heart weight of different groups. (C) Ratios of HW/TL for mice with DOX-induced heart failure after treatment with TPCD NP or TTPCD NP at 25 mg/kg via inhalation. (D) Representative M-mode echocardiography images of mouse hearts after different treatments. (E-F) LVEF and LVFS quantified by echocardiography. (G-H) The levels of MDA (G) and H_2_O_2_ (H) in heart homogenates of mice treated with different formulations. The total protein content in heart homogenates was measured by the BCA assay. (I-J) Serum levels of cTnI (I) and CK (J). Data are mean ± SD (A, n = 3; B-C,E-J, n = 5). Statistical significance was assessed by the one-way ANOVA with post-hoc LSD tests. *P < 0.05, **P < 0.01, ***P < 0.001.

At 60 h after inhalation of Cy7.5-labeled TTPCD NP (Cy7.5/TTPCD NP) in mice, *ex vivo* imaging showed significant fluorescence in the heart (Figures 8A, the left panel). Of note, inhaled Cy7.5/TTPCD NP exhibited significantly higher heart accumulation than that of Cy7.5/TPCD NP (Figure 8A, the right panel). Also, inhalation of Cy7.5/TTPCD NP resulted in notably high distribution in the liver, spleen, and kidneys (Figure S15). However, comparable fluorescence intensities in the lungs were found after inhalation of Cy7.5/TPCD NP or Cy7.5/TTPCD NP. These results suggested that TTPCD NP deposited in the lungs after inhalation could be more effectively transported across the epithelial and endothelial barriers to accumulate in the heart.

We then compared *in vivo* efficacies of two nanotherapies TPCD NP and TTPCD NP in mice with DOX-induced heart failure. Consistent with enhanced heart targeting, inhalation of TTPCD NP at 25 mg/kg more effectively inhibited the heart weight loss and reduction of the HW/TL ratio (Figure 8B-C), in comparison to the same dose of TPCD NP. Likewise, TTPCD NP prominently alleviated cardiac dysfunction as well as improved LVEF and LVFS (Figures 8D-F). In these cases, significant differences were found between TTPCD NP and TPCD NP groups. Moreover, the lowest levels of MDA and H_2_O_2_ in the myocardium as well as cTnI and CK in serum were detected in the TTPCD NP group (Figure 8G-J). Together, the mitochondrial-targeting nanotherapy TTPCD NP showed considerably potentiated therapeutic effects in amelioration of DOX-induced cardiac dysfunction, largely resulting from enhanced targeting capacities at subcellular, cellular, and tissue levels.

### TPCD NP-based pro-resolving peptide nanotherapies for prevention of heart failure in mice

**Figure 9:**
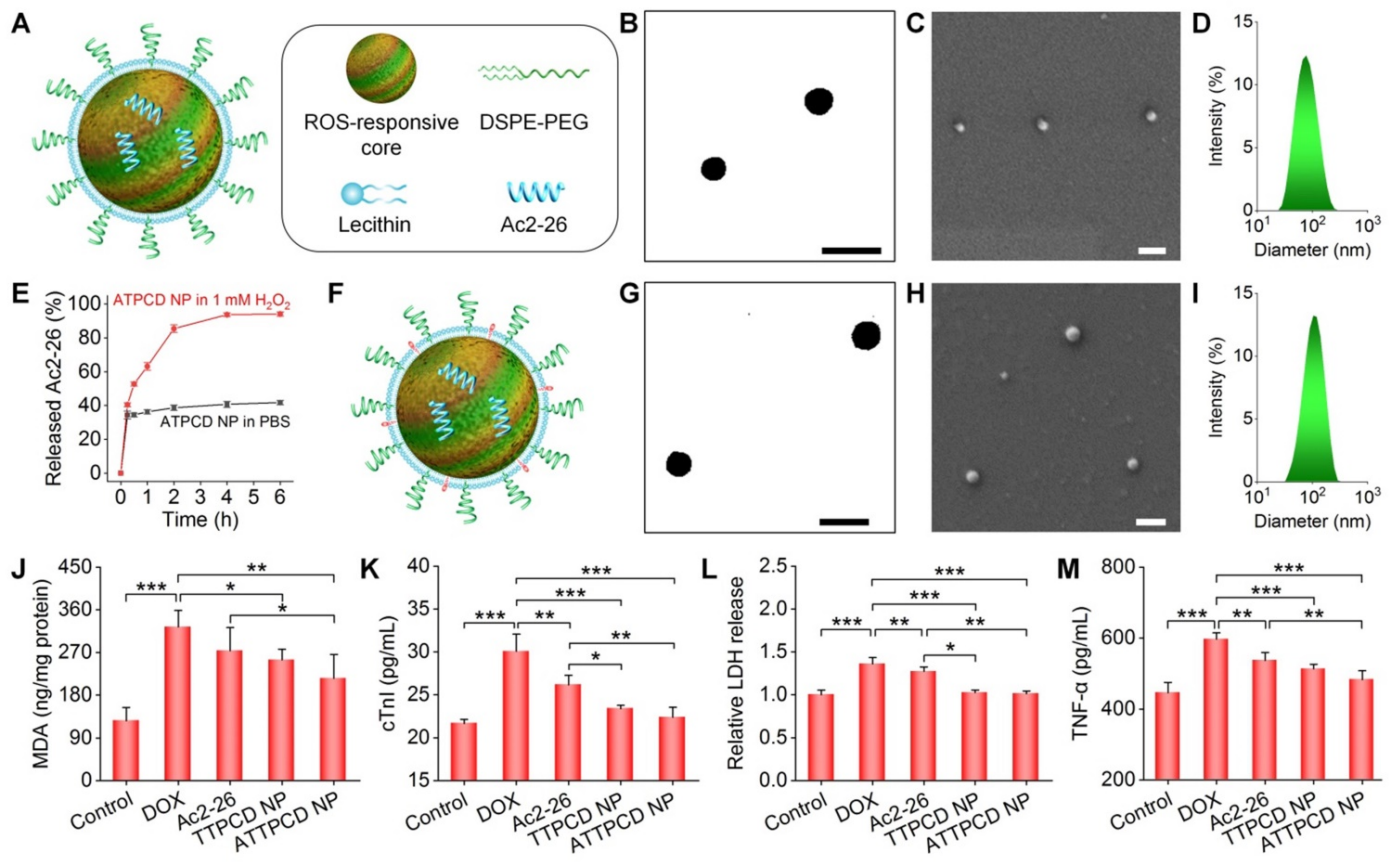
Design, engineering, and biological effects of peptide nanotherapies derived from TPCD NP. (A) Schematic of a peptide Ac2-26 nanotherapy (ATPCD NP) based on TPCD NP. (B-D) TEM (B) and SEM (C) images as well as size distribution (D) of ATPCD NP. (E) *In vitro* release profiles of FITC-Ac2-26 from ATPCD NP in 0.01 M PBS at pH 7.4 with or without 1.0 mM H_2_O_2._ (F) A sketch showing the Ac2-26 nanotherapy (ATTPCD NP) based on mitochondrial-targeting TPCD NP. (G-I) TEM (G) and SEM (H) images as well as size distribution (I) of ATTPCD NP. (J) Intracellular MDA levels in H9C2 cells after different treatments. Protein contents were measured by the BCA assay. (K-M) Expression levels of cTnI (K), LDH (L), and TNF-α (M) by H9C2 cells subjected to different treatments. Scale bars in (B-C,G-H): 200 μm. Data are mean ± SD (E, n = 3; J-M, n = 4). Statistical significance was assessed by the one-way ANOVA with post-hoc LSD tests. *P < 0.05, **P < 0.01, ***P < 0.001.

Based on the above promising findings, we reasonably considered that TPCD NP can serve as a bioactive nanoplatform for ROS-responsive delivery of molecular therapeutics. Given the crucial role of inflammation in the development of heart failure 43, a pro-resolving peptide Ac2-26 was used as a model anti-inflammatory drug in this conceptual proof study. Ac2-26-containing TPCD NPs (*i.e.*, ATPCD NP) were also prepared by the nanoprecipitation/self-assembly method as aforementioned (Figure 9A). Spherical NPs with well-defined morphology and narrow size distribution were obtained (Figure 9B-D), and the average diameter of ATPCD NP was 104 nm. *In vitro* tests clearly showed ROS-responsive release of Ac2-26 from ATPCD NP, as indicated by considerably rapid release at 1 mM H_2_O_2_ (Figure 9E). This is well agreeing with ROS-sensitive properties of TPCD NP 49. Then a mitochondrial targeting Ac2-26 nanotherapy was developed (Figure 9F), which was defined as ATTPCD NP. Similar to other nanotherapies, ATTPCD NP also displayed spherical shape and narrow size distribution (Figure 9G-I), with the mean diameter of 109 nm.

**Figure 10:**
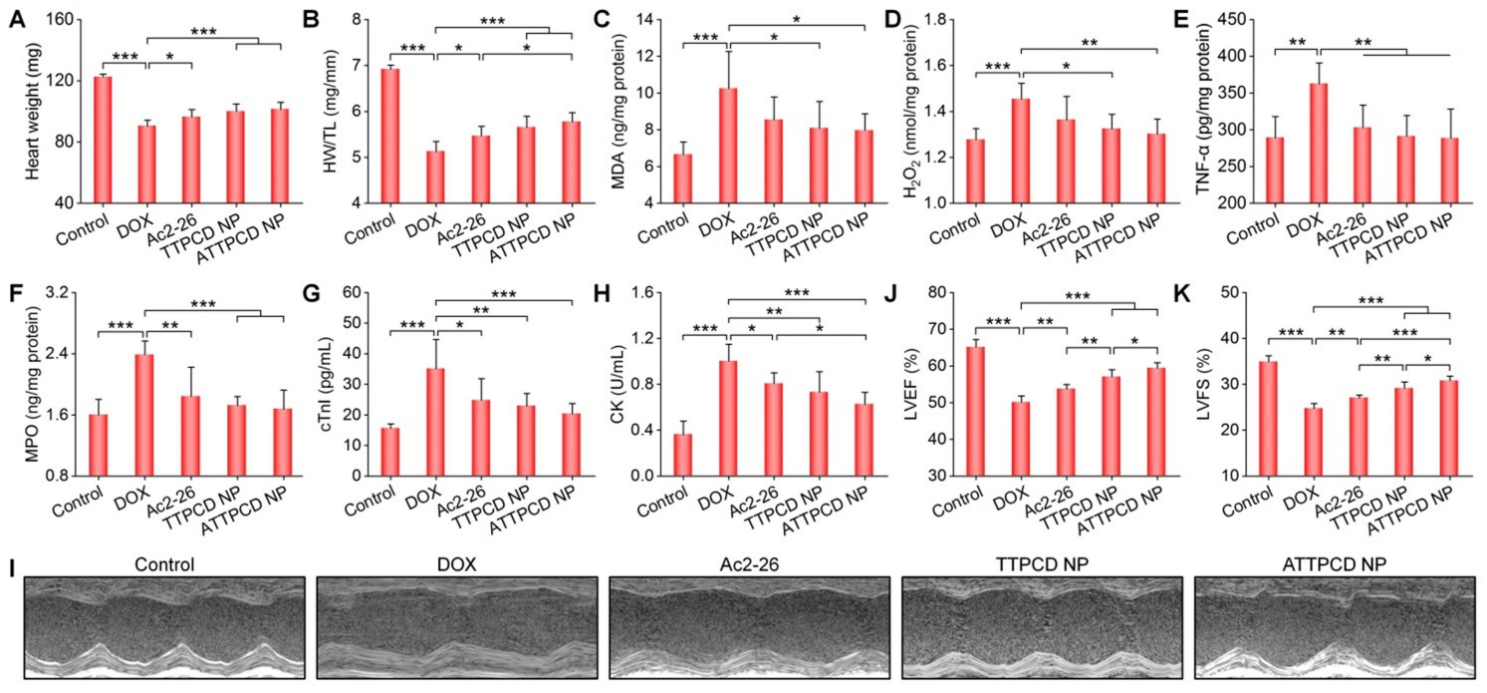
Therapeutic effects of the mitochondrial-targeting peptide nanotherapy ATTPCD NP after inhalation delivery in mice with DOX-induced heart failure. (A) The heart weight of different groups. (B) The ratios of HW/TL for different groups. (C-F) The levels of MDA (C), H_2_O_2_ (D), TNF-α (E), and MPO (F) in tissue homogenates of hearts isolated from mice treated with different formulations. Total protein contents in heart homogenates were measured by the BCA assay. (G-H) Serum levels of cTnI (G) and CK (H). (I) Representative M-mode echocardiography images of mouse hearts after different treatments. (J-K) LVEF and LVFS quantified by echocardiography. Data are mean ± SD (n = 5). Statistical significance was assessed by the one-way ANOVA with post-hoc LSD tests. *P < 0.05, **P < 0.01, ***P < 0.001.

Subsequently, *in vitro* studies were performed to evaluate activities of ATTPCD NP in H9C2 cells. Compared to free Ac2-26 and TTPCD NP, ATTPCD NP more effectively protected H9C2 cells from DOX-induced injury, as implicated by notably decreased levels of MDA, cTnI, LDH, and TNF-α (Figure 9J-M). Further, we examined *in vivo* efficacy of ATTPCD NP in mice with DOX-induced cardiac dysfunction. Inhalation of ATTPCD NP inhibited the cardiac weight loss and decrease in the HW/TL ratio to a much more extent than those of Ac2-26 and TTPCD NP (Figure 10A-B). Likewise, therapy with ATTPCD NP more effectively decreased the expression levels of MDA, H_2_O_2_, MPO, and TNF-α in heart tissues (Figure 10C-F), as compared to both Ac2-26 and TTPCD NP groups. In addition, serum levels of cTnI and CK were potently reduced after treatment with ATTPCD NP (Figure 10G-H). Consistently, ATTPCD NP more significantly alleviated cardiac dysfunction as well as improved LVEF and LVFS than those of Ac2-26 and TTPCD NP (Figure 10I-K). Collectively, these results demonstrated that TTPCD NP can be used as an effective bioactive nanoplatform to substantially potentiate anti-inflammatory effects of loaded Ac2-26.

### Safety studies on TPCD NP

Finally, both *in vitro* and *in vivo* experiments were performed to examine the safety profile of TPCD NP. We first tested *in vitro* cytotoxicity of TPCD NP in different cells associated with absorption and accumulation of inhaled nanotherapies. After 12 or 24 h of incubation with different doses of TPCD NP, high cell viability was found for H9C2 cells, A549 cells, HUVECs, and RAW264.7 macrophages (Figure S16). Even at the highest dose of 1000 µg/mL, cell viability was still above 80% in most cases. These results implied that TPCD NP displayed low cytotoxicity.

Since TPCD NP is expected to be delivered via inhalation, possible side effects after inhalation at low (62.5 mg/kg/day) and high (125 mg/kg/day) doses were tested in mice. During 7 days of continuous inhalation, no significant changes were found for the mouse body weight (Figure S17A). After treatment, we did not find significant variations in the major organ index and lung wet/dry weight ratios (Figure S17B-C). Moreover, no significant changes were detected for two typical proinflammatory cytokines of TNF-α and IL-1β (Figure S17D-E) in bronchoalveolar lavage fluid. Likewise, TPCD NP-treated mice exhibited no significant abnormal changes in the levels of representative hematological parameters and biomarkers relevant to hepatic and kidney functions (Figure S18). Further, assessment of H&E-stained sections revealed no discernable injuries in the trachea and other major organs (Figure S19). These results collectively suggested that TPCD NP displayed good safety profile after inhalation at doses at least 5-fold higher than those used in therapeutic studies.

## Discussion

Currently, new therapies and creative drug delivery strategies are still required for effective and safe treatment of heart failure. Whereas inhalation delivery is promising for noninvasive delivery of molecular therapies and nanotherapies for cardiovascular diseases 19, 25, 36, pulmonary translocation and heart targeting mechanisms of inhaled NPs remain unclear. In this study, we attempt to explore heart targeting mechanisms after inhalation using a nanotherapy based on bioactive NPs (*i.e.*, TPCD NP). Our results showed that inhaled TPCD NP can avoid removal by the airway mucociliary escalator, thereby affording effective deposition in the lungs in a time-dependent manner. The maximal lung accumulation of Cy7.5/TPCD NP was almost achieved within 12 h after inhalation. Similarly, Cy7.5/TPCD NP displayed a gradually increased heart accumulation, with a notable distribution at 12 h after inhalation. Whereas previous studies have demonstrated translocation of inhaled NPs across the air-blood barrier 19, 25, 31, 76, the exact route of intrapulmonary translocation has not been elucidated thus far. Both fluorescence observation and flow cytometric quantification indicated that pulmonary absorption and subsequent translocation of TPCD NP were mainly mediated by alveolar epithelial cells and endothelial cells. By contrast, considerably low uptake of TPCD NP was detected in alveolar macrophages, suggesting that lung macrophages only displayed negligible effects on absorption and clearance of TPCD NP. In addition, flow cytometric analysis showed a very low distribution of TPCD NP in typical lymphocytes. Accordingly, lymphatic transport is not a major pathway responsible for the heart accumulation of inhaled TPCD NP. In combination with the finding that the dominant alveolocapillary permeability barrier is due to the epithelial cells 40, we can conclude that pulmonary translocation of inhaled TPCD NP is mainly dominated by epithelial and endothelial cells via the caveolae-mediated transcytosis and/or tight junction-regulated paracellular transport. To a large degree, thus absorbed NPs in pulmonary capillaries can be further transported to the heart via the pulmonary circuit, since this is the shortest path of circulation between the heart and lungs.

Further, we demonstrated therapeutic advantages of nanotherapies based on intrinsically antioxidative and anti-inflammatory NPs in the targeted treatment of heart failure by inhalation delivery. The mentioned nanotherapy TPCD NP can be easily formulated from a broad-spectrum ROS-scavenging material derived from a cyclic oligosaccharide 49. *In vitro* experiments revealed excellent ROS-scavenging capability of TPCD NP. By efficiently scavenging intracellular ROS, TPCD NP inhibited ROS-induced lipid peroxidation and DOX-induced cell damage in cardiomyocytes. After inhalation, TPCD NP effectively reversed the heart weight loss caused by DOX. More importantly, TPCD NP notably reduced cardiac dysfunction and inhibited myocardial injury in mice. Compared with DEX, a clinically used cardioprotective agent, TPCD NP exhibited more significant therapeutic effects in the treatment of DOX-induced heart failure in mice. By surface decoration of TPCD NP with STPP, a mitochondrial targeting nanotherapy TTPCD NP was obtained, which showed significantly enhanced cellular uptake and antioxidative activity in cardiomyocytes. Moreover, inhaled TTPCD NP displayed considerably higher heart accumulation than that of TPCD NP. These results demonstrated that functionalization with STPP can simultaneously enhance TPCD NP targeting capacity at three different levels from the heart tissue, cardiomyocytes, to subcellular mitochondria. Correspondingly, notably potentiated cardiac protection effects were achieved by TTPCD NP. In addition, TTPCD NP can serve as a bioactive nanovehicle for heart-specific delivery of anti-inflammatory molecular therapeutics, affording notably enhanced efficacies. It is worth noting that male mice were used in our *in vivo* therapeutic experiments. Previous studies have demonstrated that female sex protected against DOX-induced cardiac dysfunction in rats of different strains 77-80. Possible mechanisms, such as mitochondrial dysfunction, energy metabolism, cardiolipin remodeling, and estrogen regulation have been proposed to explain sexual dimorphism of DOX-induced heart failure. However, the exact mechanisms remain to be fully explored. Also, additional studies are necessary to address whether TPCD-derived nanotherapies are effective for prevention of heart failure in female rodents.

Of note, our preliminary studies showed that TPCD NP is safe after inhalation delivery at doses at least 5-fold higher than that used for *in vivo* therapy. Compared with previously developed nanotherapies for heart failure 16, 19, 22, our nanotherapies based on TPCD NP have multiple advantages, such as desirable efficacy, good safety, easy production, and low cost, thereby deserving further intensive development. Importantly, we may reasonably expect that efficacy of TPCD NP nanotherapies can be additionally enhanced by loading other drugs to achieve synergistic effects.

## Conclusions

In summary, we demonstrated that inhaled NPs can accumulate in the heart by transport across the lung epithelial and endothelial barriers through the pulmonary circulation, mainly via the transcellular and/or paracellular pathways. Based on this pulmonary circulation-mediated heart targeting strategy, inhalation of an antioxidative and anti-inflammatory nanotherapy TPCD NP effectively prevented heart failure in a mouse model induced by DOX at low doses. By surface engineering with a functional moiety, mitochondrial-targeting, cellular uptake, and heart accumulation capacities of TPCD NP were considerably enhanced, thereby affording additionally potentiated therapeutic effects in mice with DOX-induced heart failure. It is worth noting that TPCD NP can also serve as a bioactive nanovehicle for heart-specific delivery of anti-inflammatory molecular therapeutics, resulting in remarkably potentiated *in vivo* efficacies. Moreover, preliminary tests revealed good safety profile of inhaled TPCD NP. Consequently, pulmonary circulation-mediated heart targeting of NPs is an intriguing approach for therapy of heart diseases, while TPCD-based nanotherapies hold great promise for the treatment of oxidative stress-associated acute or chronic heart diseases such as heart failure, myocardial ischemia-reperfusion injury, arrhythmia, and myocardial hypertrophy.

## Supplementary Material

Supplementary materials and methods, figures.
