# Supplementary material for: Pulmonary circulation-mediated heart targeting for the prevention of heart failure by inhalation of intrinsically bioactive nanoparticles
Source: Theranostics. 2021 Jul 25;11(17):8550–69. doi: 10.7150/thno.61875 (PMC8343995; doi:10.7150/thno.61875)
Supplement: Supplementary file 1 — Supplementary materials and methods, figures. [file thnov11p8550s1.pdf]

Supplementary Materials for

Pulmonary circulation-mediated heart targeting for the prevention of heart failure by inhalation of intrinsically bioactive nanoparticles

Chao Liu[1], Liyuan Chen[1], Yongchang Ma[2], Kaiyao Hu[2], Peng Wu[2], Lina Pan[1], Haiyan Chen[1], Lanlan Li[2], Houyuan Hu[1,]*, Jianxiang Zhang[2,3,]*

1. Department of Cardiology, Southwest Hospital, Third Military Medical University (Army Medical University), Chongqing 400038, China
2. Department of Pharmaceutics, College of Pharmacy, Third Military Medical University (Army Medical University), Chongqing 400038, China
3. State Key Lab of Trauma, Burn and Combined Injury, Institute of Combined Injury, Third Military Medical University (Army Medical University), Chongqing 400038, China

*Corresponding authors:
Houyuan Hu, PhD, Prof.
Department of Cardiology
Southwest Hospital
Third Military Medical University (Army Medical University)
Chongqing 400038, China
E-mail: houyuanhu@hotmail.com

Jianxiang Zhang, PhD, Prof.
Department of Pharmaceutics
College of Pharmacy
Third Military Medical University (Army Medical University)
Chongqing 400038, China
E-mail: jxzhang1980@gmail.com, jxzhang@tmmu.edu.cn

Supplementary materials and methods

Materials

Tempol (Tpl), 4-(hydroxymethyl)phenylboronic acid pinacol ester (PBAP), β-cyclodextrin (β-CD), 4-dimethylaminopyridine (DMAP), 1,1-carbonyldiimidazole (CDI), anhydrous dimethylformamide (DMF), anhydrous dimethylsulfoxide (DMSO), anhydrous dichloromethane (DCM), 2,2-diphenyl-2-picrylhydrazyl (DPPH), 4',6-diamidino-2-phenylindole (DAPI), triphenylphosphine (TPP), and dihydroethidium (DHE) were purchased from Sigma-Aldrich (USA). Lecithin was supplied by Tokyo Chemical Industry Co., Ltd (Tokyo, Japan). 1,2-Distearoyl-sn-glycero-3-phosphoethanolamine-N-[methoxyl(polyethylene glycol)-2000] (DSPE-PEG) was obtained from Avanti Polar Lipids, Inc. (USA). Ac2-26 (Ac-AMVSEFLKQAWFIENEEQEYVQTVK) and FITC-labeled Ac2-26 (FITC-Ac2-26) were synthesized by ChinaPeptides Co., Ltd. (China). Penicillin, streptomycin, fetal bovine serum (FBS), and Dulbecco's modified eagle medium (DMEM) were provided by Gibco (Waltham, USA). Cyanine5 NHS ester (Cy5) and cyanine7.5 NHS ester (Cy7.5) were obtained from Lumiprobe (USA). 2',7'-Dichlorofluorescein diacetate (DCFH-DA), BCA protein assay kit, and lactate dehydrogenase (LDH) activity assay kit were obtained from Beyotime Biotechnology (China). Doxorubicin (DOX) and dexrazoxane (DEX) were purchased from MedChemExpress (USA). ELISA kits for cardiac troponin I (cTnI), creatine kinase-MB isoenzyme (CK-MB), and malondialdehyde (MDA) were obtained from Elabscience Biotechnology (China). Myeloperoxidase (MPO), tumor necrosis factor (TNF)-α, interleukin (IL)-1β, and cell counting kit (CCK)-8 were purchased from Boster Biological Technology, Ltd (China). Creatine kinase (CK) assay kit was purchased from Nanjing Jiancheng Bioengineering Institute (China). Red hydrogen peroxide and MitoTracker Green FM were purchased from Invitrogen (USA). Anti-mouse CD90.2 antibody, anti-mouse CD45R antibody, anti-mouse NK-1.1 antibody, anti-mouse Ly6G antibody, anti-mouse Ly6C antibody, anti-mouse CD326 antibody, and anti-mouse CD31 antibody were purchased from BD Biosciences (USA). Anti-mouse CD49b antibody was purchased from eBioscience (USA). Anti-mouse CD11c antibody, anti-mouse F4/80 antibody, and anti-mouse CD11b antibody were obtained from Biolegend (USA). Anti-CD31 rabbit monoclonal antibody, anti-CD68 rabbit polyclonal antibody, and the secondary antibody Alexa Fluor 488-conjugated goat anti-rabbit IgG were obtained from Wuhan Servicebio Technology Co., Ltd (China).

Synthesis of a reactive oxygen species (ROS)-scavenging material based on β-CD

A ROS-scavenging material (defined as TPCD) was synthesized by sequentially conjugating Tpl and PBAP onto β-CD according to our previously established method [1]. First, Tpl-conjugated β-CD (TCD) was prepared. Specifically, Tpl (0.51 g, 2.95 mmol) was dissolved in 7.5 mL of anhydrous DCM, and then CDI (0.955 g, 5.9 mmol) was added. After reaction at 30°C for 45 min, the reaction mixture was washed once with 5 mL of distilled water, and then washed twice with 5 mL of saturated NaCl solution. The obtained material was dried with Na_2SO_4, and the organic solvent was removed by rotary evaporation to obtain CDI-activated Tpl (0.825g). CDI-activated Tpl (0.825 g), β-CD (1.175 g, 1.035 mmol), and DMAP (0.565 g, 4.65 mmol) were dissolved in 10 mL of anhydrous DMSO, and stirred at 30°C under nitrogen. After reaction for 24 h, a light pink precipitate was obtained by adding methanol and diethyl ether. After centrifugation and lyophilization, TCD was obtained.

Then CDI-activated PBAP was synthesized. Briefly, PBAP (0.555 g) and CDI (0.765 g) were added to 7.5 mL of anhydrous DCM, and stirred at 30°C for 45 min under nitrogen. The organic phase was washed with 10 mL of deionized water and 10 mL of saturated NaCl solution, dried over Na_2SO_4, and concentrated to obtain CDI-activated PBAP.

Subsequently, TCD (0.24 g), CDI-activated PBAP (0.83 g), and DMAP (0.53 g) were dissolved in 10 mL of anhydrous DMSO under nitrogen at 30°C for 48 h. At the end of the reaction, a light pink product was precipitated with deionized water. The solvent was removed by centrifugation, and the product TPCD was dried in a vacuum drying oven.

Synthesis of stearyl-TPP

Stearyl-TPP (*i.e.*, STPP) was synthesized according to the method previously reported [2]. Specifically, stearyl bromide (0.5 g, 1.5 mmol) and triphenylphosphine (0.415 g, 1.6 mmol) were dissolved in acetonitrile (12.5 mL). The resulting solution was heated and refluxed in nitrogen atmosphere overnight. The reaction was cooled to room temperature and concentrated under reduced pressure. The resulting oil was washed with hexanes (2 × 10 mL) and diethyl ether (2 × 7.5 mL), and finally dried to obtain a white solid.

Characterization of nanoparticles

Mean sizes, size distribution profiles, and zeta-potential values of different NPs were measured using a Malvern Zetasizer (Nano ZS90, Malvern Instruments, UK) at 25°C. The morphology of NPs was observed by transmission electron microscopy (TEM) using a JEM-1400 microscope (JEOL, Tokyo, Japan). Scanning electron microscopy (SEM) was performed with a FIB-SEM microscope (Crossbeam 340, Zeiss).

Determination of ROS-scavenging capability of TPCD NP

Free radical scavenging capability of TPCD NP was measured according to our previously established method [1]. In brief, 1 mL of fresh solution of 2,2-diphenyl-1-picrylhydrazyl radical (DPPH•) at 100 μg/mL was incubated with 2 mL of methanol solutions containing different concentrations of TPCD NP (from 0, 0.063, 0.125, 0.25, 0.5, 1.0, to 2.0 mg/mL) for 30 min in dark. Absorbance at 517 nm was then recorded by UV-visible spectroscopy and eliminated DPPH• was calculated.

Superoxide anion-scavenging capability of TPCD NP was also evaluated. To this end, different concentrations of TPCD NP were incubated with excess superoxide anion produced by the xanthine/xanthine oxidase system at 37°C for 40 min. The remaining superoxide anion was detected with the superoxide anion detection kit (Nanjing Jiancheng Bioengineering Institute, China). Then the superoxide anion elimination ability of TPCD NP was calculated.

To examine the H_2O_2-scavenging capacity, aqueous solutions containing different concentrations of TPCD NP (2 mL) were incubated with 2 mL of PBS containing 50 mM H_2O_2 at 37°C for 24 h. The concentration of remaining H_2O_2 was determined by using a hydrogen peroxide detection kit (Nanjing Jiancheng Bioengineering Institute, China), and eliminated H_2O_2 was calculated.

***In vitro* cytotoxicity of various nanoparticles**

A549 cells, HUVECs, RAW264.7 macrophages, and H9C2 cells were cultured in 96-well plates in 100 μL of culture medium. A549 cells, HUVECs, and RAW264.7 cells were incubated at 1.0×10^4 cells per well, while H9C2 cells were at 5×10^3 per well. The cells were treated with different doses of NPs for different time periods. The cell viability was quantified by CCK-8 assay.

Histological analysis

Hearts were fixed in 4% paraformaldehyde. Then paraffin-embedded sections were prepared and stained with hematoxylin and eosin (H&E).

References

1. Li L, Guo J, Wang Y, Xiong X, Tao H, Li J, et al. A broad-spectrum ros-eliminating material for prevention of inflammation and drug-induced organ toxicity. Adv Sci. 2018; 5: 1800781.
2. Marrache S, Dhar S. Biodegradable synthetic high-density lipoprotein nanoparticles for atherosclerosis. Proc Natl Acad Sci U S A. 2013; 110: 9445-50.

Supplementary results

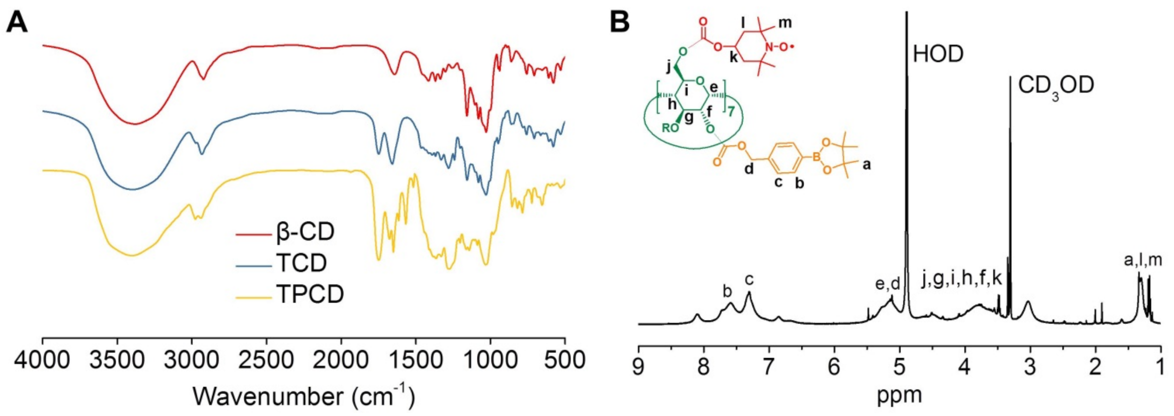

Figure S1. Characterization of a ROS-eliminating bioactive material TPCD. (A-B) FT-IR (A) and ^{1}H NMR (B) spectra of different materials.

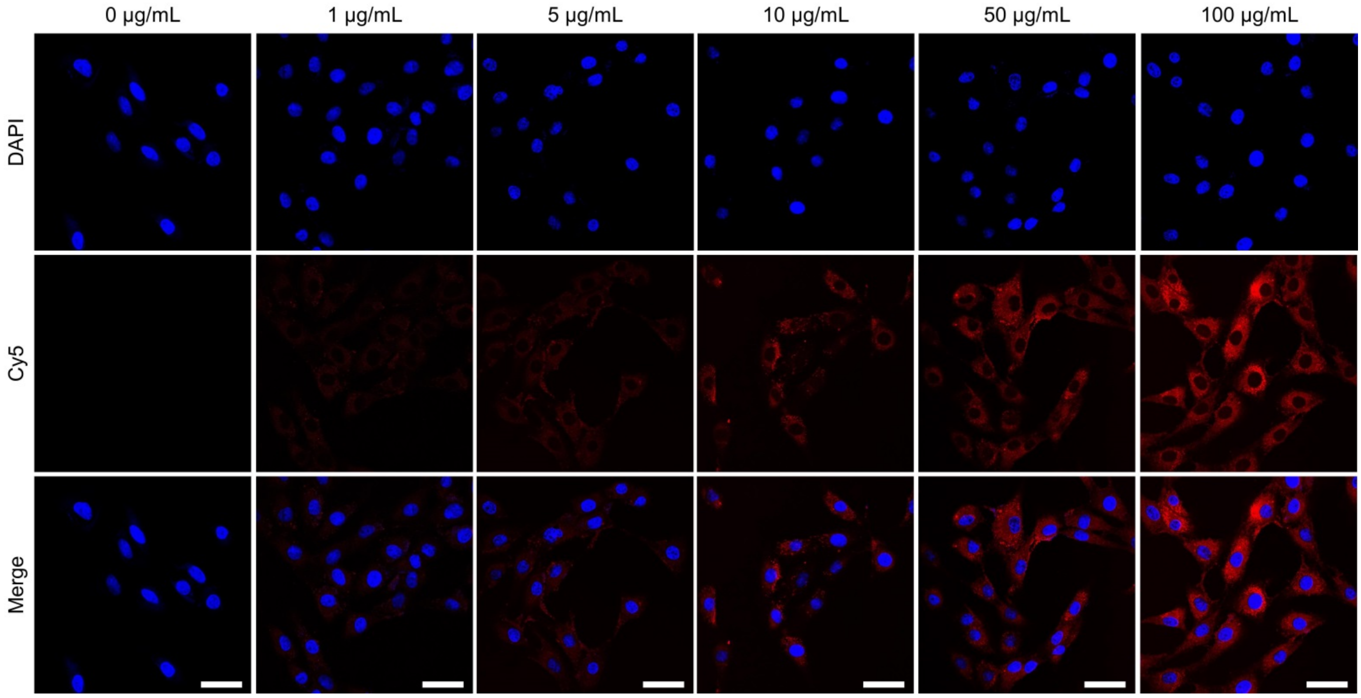

Figure S2. Confocal microscopy images showing dose-dependent cellular uptake of Cy5/TPCD NP. H9C2 cells were incubated with different doses of Cy5/TPCD NP for 2 h, then nuclei were stained with DAPI. Scale bars, 40 μm.

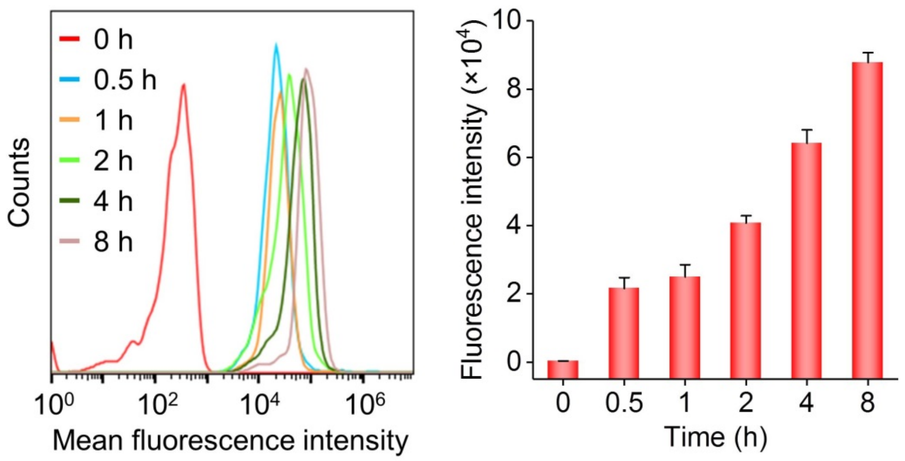

Figure S3. *In vitro* cellular uptake of TPCD NP in neonatal rat ventricular myocytes (NRVMs). Typical flow cytometric curves (left) and quantitative analysis (right) of time-dependent cellular uptake of Cy5/TPCD NP in NRVMs. Data are mean ± SD (n = 3).

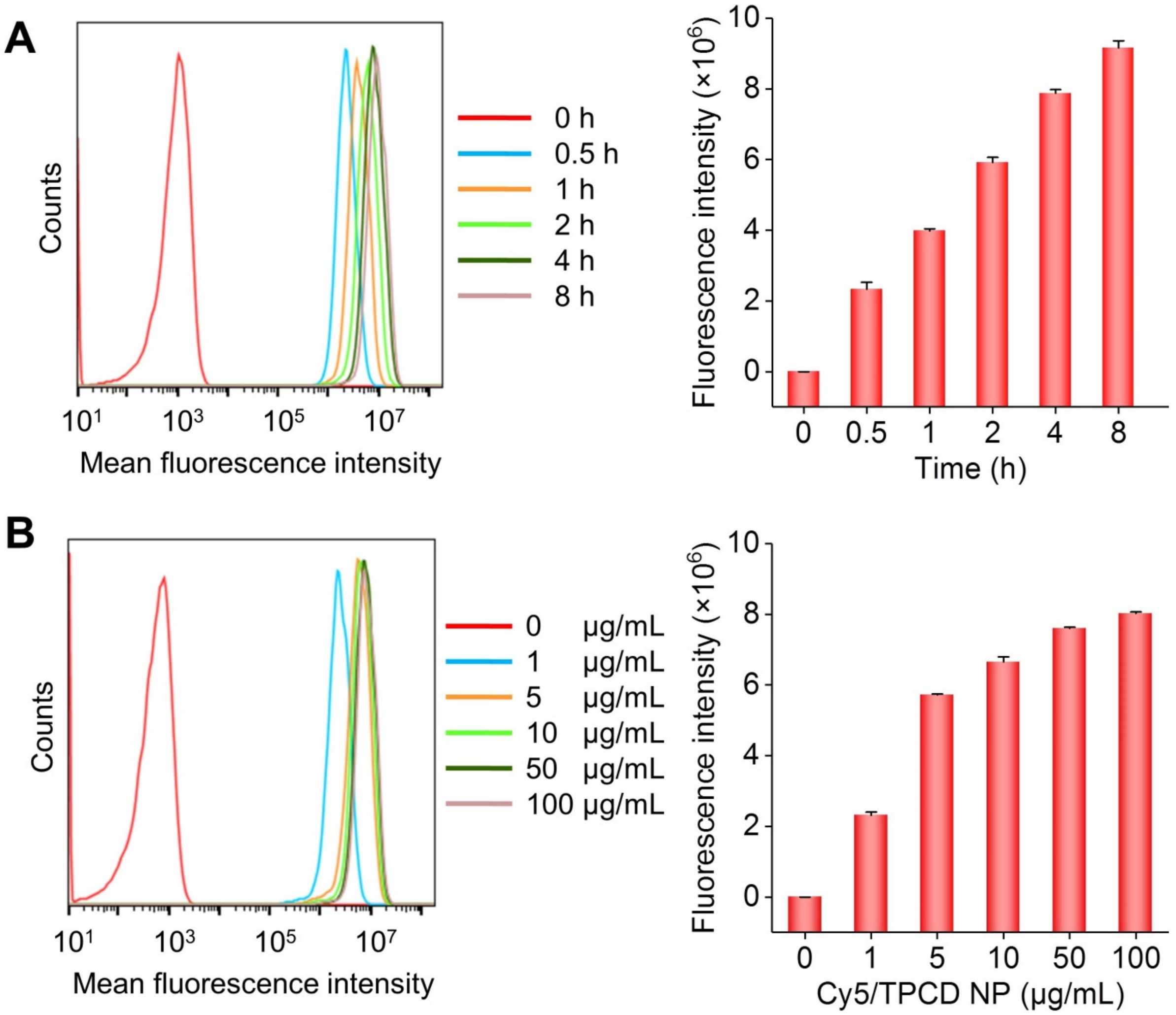

Figure S4. Flow cytometric analysis of cellular uptake profiles of TPCD NP in A549 human lung epithelial cells. (A-B) Flow cytometric curves (left) and quantitative analysis (right) of time (A) and dose (B) dependent cellular uptake of Cy5/TPCD NP. In the time-dependent study, the concentration of Cy5/TPCD NP was 50 μg/mL, while cells were incubated with Cy5/TPCD NP for 2 h in the dose-dependent experiments. Data are mean ± SD (n = 3).

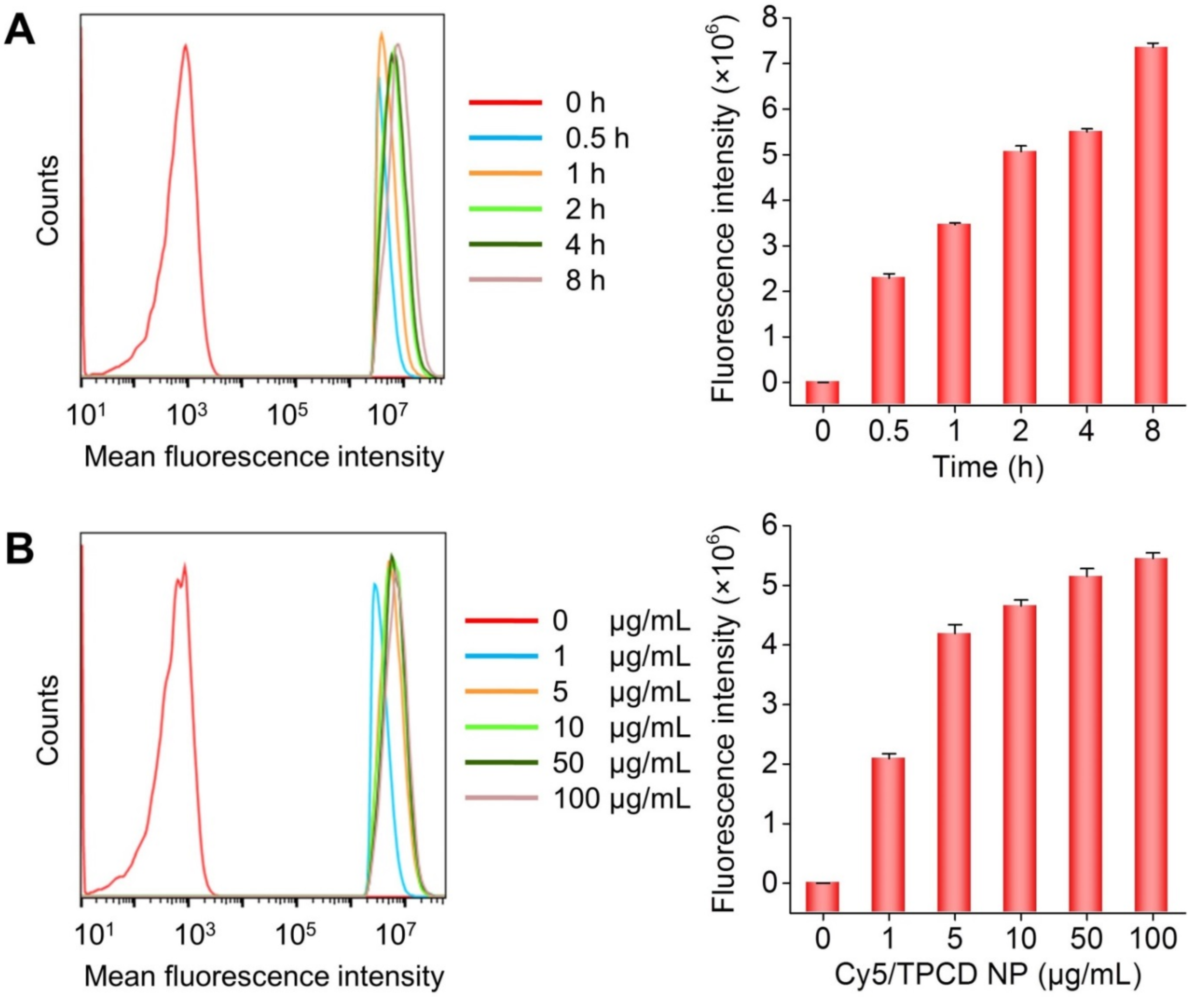

Figure S5. Cellular uptake profiles of TPCD NP in HUVECs. (A-B) Flow cytometric curves (left) and quantitative analysis (right) of time (A) and dose (B) dependent cellular uptake of Cy5/TPCD NP. In the time-dependent study, the concentration of Cy5/TPCD NP was 50 μg/mL, while cells were incubated with Cy5/TPCD NP for 2 h in the dose-dependent experiments. Data are mean ± SD (n = 3).

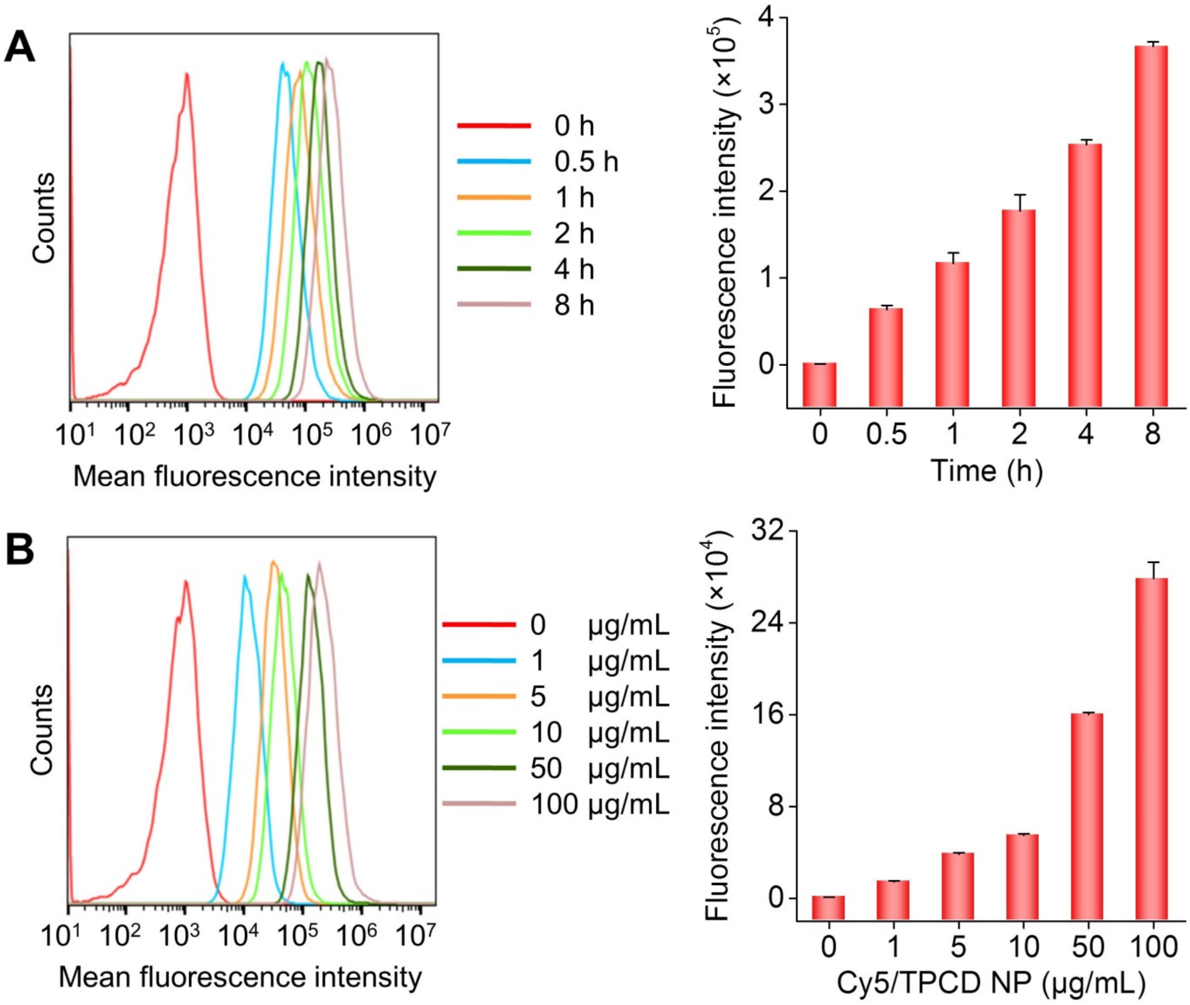

Figure S6. Cellular uptake profiles of TPCD NP in RAW264.7 murine macrophages. (A-B) Flow cytometric curves (left) and quantitative analysis (right) of time (A) and dose (B) dependent cellular uptake of Cy5/TPCD NP. In the time-dependent study, the concentration of Cy5/TPCD NP was 50 μg/mL, while cells were incubated with Cy5/TPCD NP for 2 h in the dose-dependent experiments. Data are mean ± SD (n = 3).

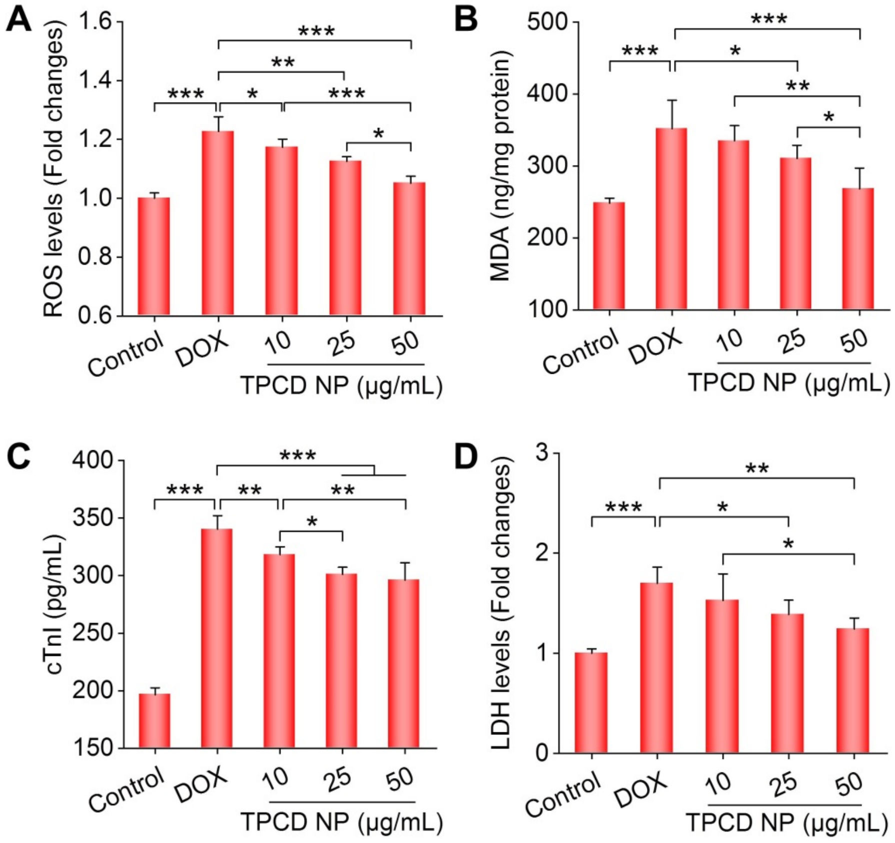

Figure S7. *In vitro* biological effects of TPCD NP in neonatal rat ventricular myocytes (NRVMs). (A-B) Intracellular ROS (A) and MDA (B) levels after different treatments. The protein content was measured by the BCA assay. (C-D) Expression levels of cTnI (C) and LDH (D). The control group was only treated with culture medium. Cells in the DOX group were treated with DOX alone. TPCD NP groups were pretreated with different doses of TPCD NP for 2 h, followed by stimulation with DOX for 24 h. Data are mean ± SD (n = 4). Statistical significance was assessed by the one-way ANOVA. *P < 0.05, **P < 0.01, ***P < 0.001.

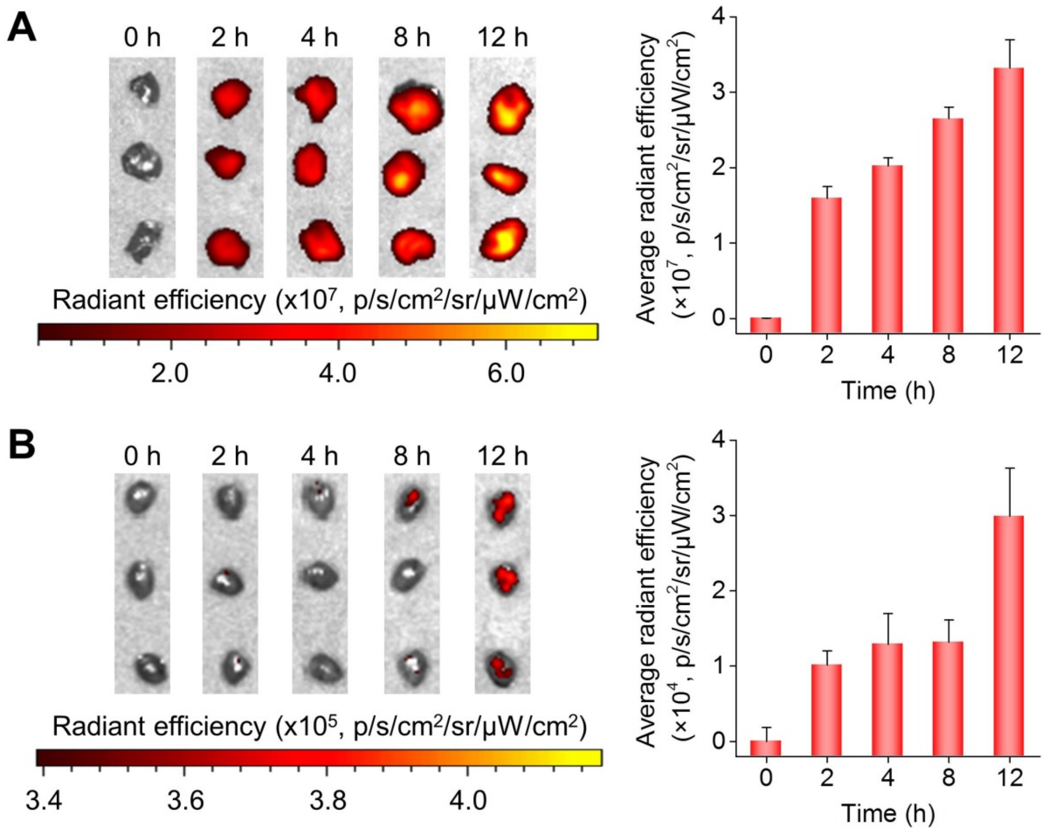

Figure S8. Accumulation of Cy7.5/TPCD NP in lungs and hearts after inhalation delivery. (A-B) *Ex vivo* fluorescence images (left) and quantitative analysis (right) show the distribution of Cy7.5/TPCD NP in lungs (A) and hearts (B) of mice after inhalation for different periods of time. Data are mean ± SD (n = 3).

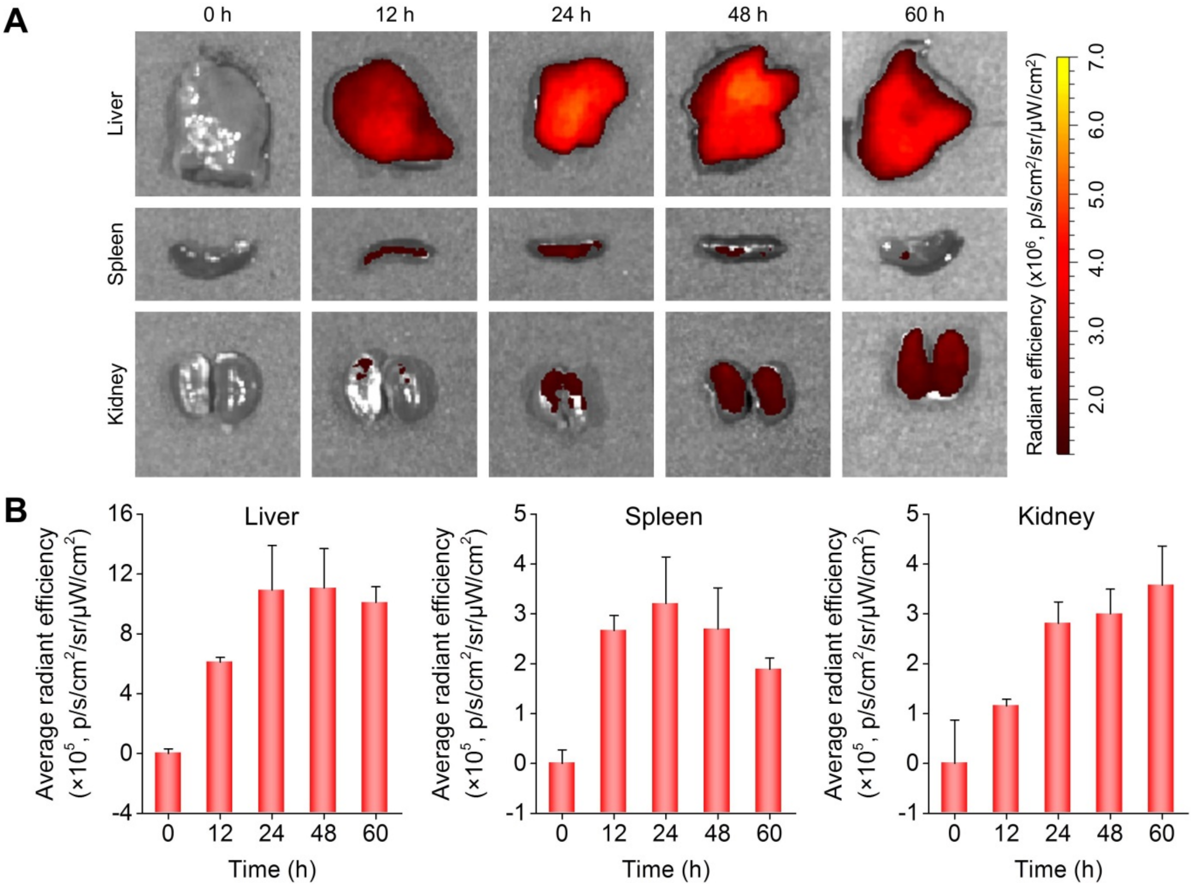

Figure S9. Accumulation of Cy7.5/TPCD NP in major organs after inhalation. (A-B) *Ex vivo* fluorescence images (A) and quantitative analysis (B) show the distribution of Cy7.5/TPCD NP in the liver, spleen, and kidneys of mice after inhalation for different periods of time. Data are mean ± SD (n = 3).

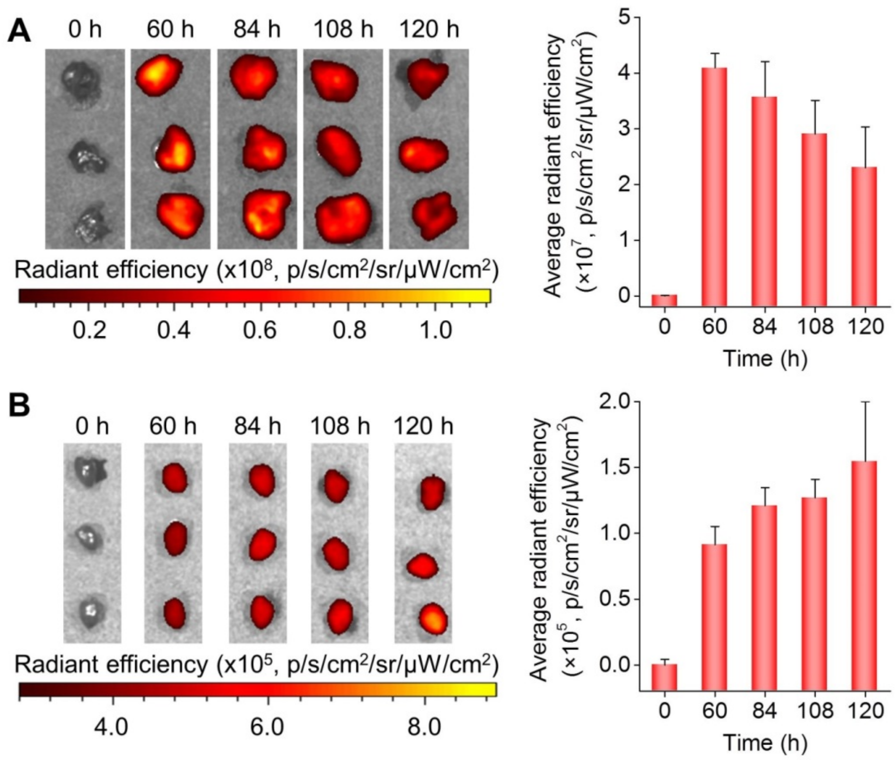

Figure S10. Accumulation of Cy7.5/TPCD NP in lungs and hearts after inhalation delivery. (A-B) *Ex vivo* fluorescence images (left) and quantitative analysis (right) show the distribution of Cy7.5/TPCD NP in lungs (A) and hearts (B) of mice after inhalation for different periods of time. Data are mean ± SD (n = 3).

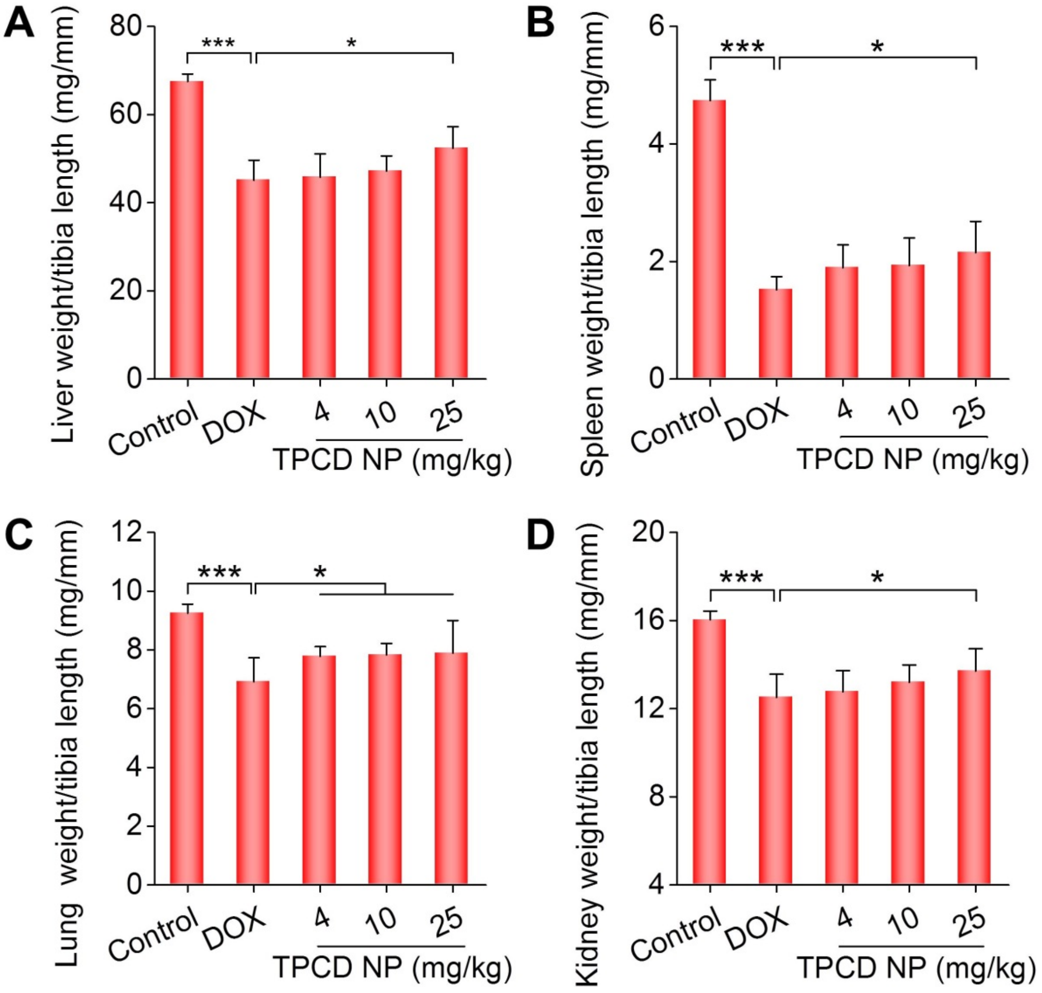

Figure S11. The ratios of organ weight to tibia length. (A-D) The ratios of liver weight to tibia length (A), spleen weight to tibia length (B), lung weight to tibia length (C), and kidney weight to tibia length (D) after inhalation of different doses of TPCD NP in mice with DOX-induced cardiac dysfunction. Data are mean ± SD (n = 5). *P < 0.05, ***P < 0.001.

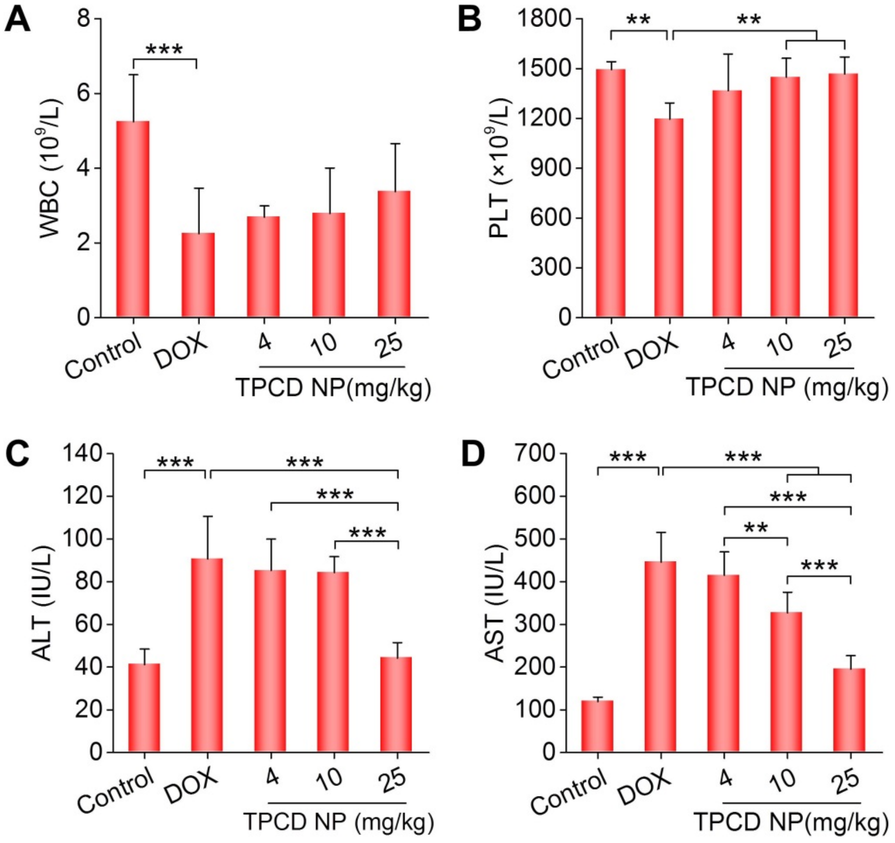

Figure S12. Evaluations of *in vivo* therapeutic effects after inhalation of different doses of TPCD NP in mice with DOX-induced cardiac dysfunction. (A-B) Levels of WBC (A) and PLT (B). (C-D) Serum levels of ALT (C) and AST (D). WBC, white blood cells; PLT, platelets; ALT, alanine aminotransferase; AST, aspartate aminotransferase. Data are mean ± SD (n = 5). $^{**}P < 0.01$, $^{***}P < 0.001$.

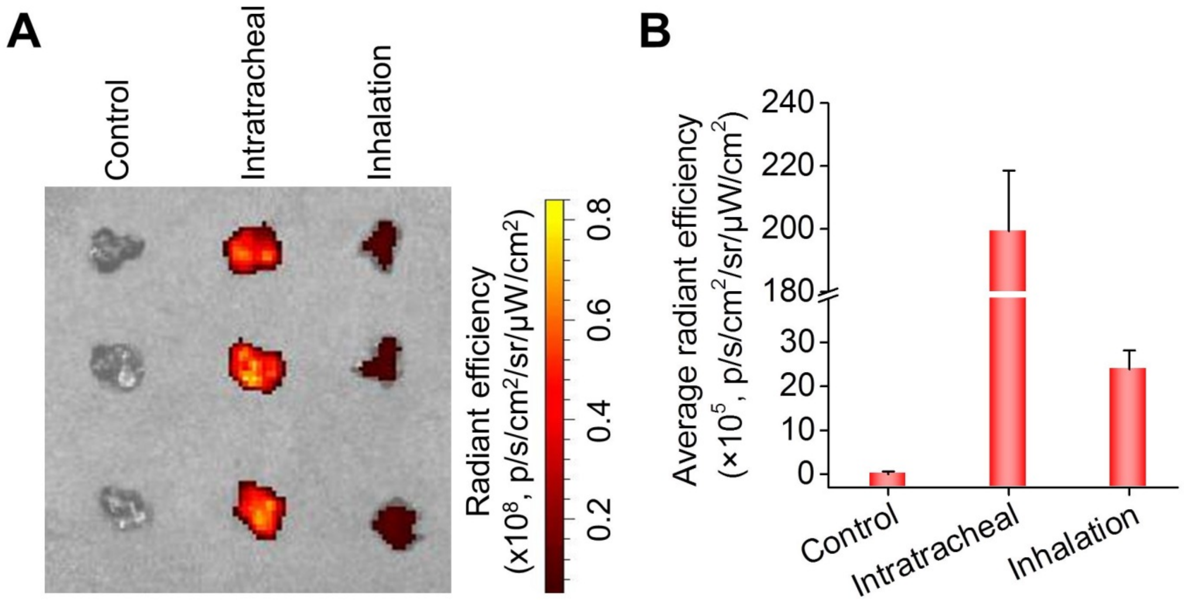

Figure S13. Comparison of delivery efficiency of inhalation with intratracheal administration. (A-B) Representative *ex vivo* images (A) and quantitative analysis (B) of Cy7.5/TPCD NP fluorescence intensities in the lungs of mice after direct intratracheal instillation via a Microsprayer Aerosolizer or inhalation after nebulization. Data are mean ± SD (n = 3).

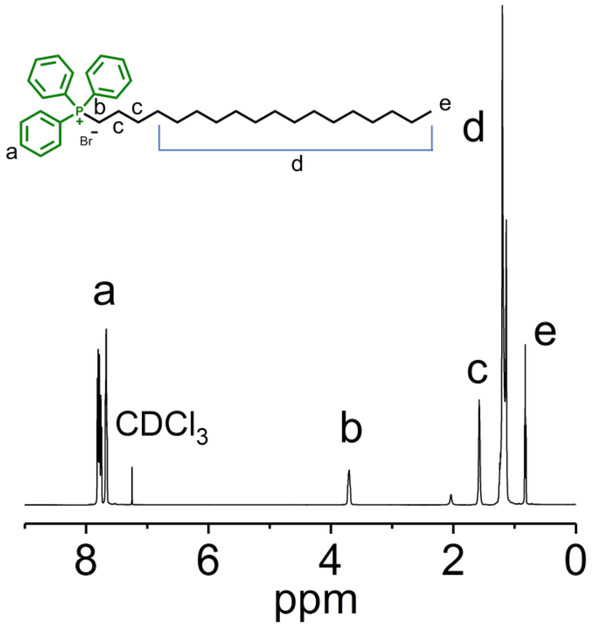

Figure S14. ^{1}H NMR spectrum of STPP in $CDCl_3$.

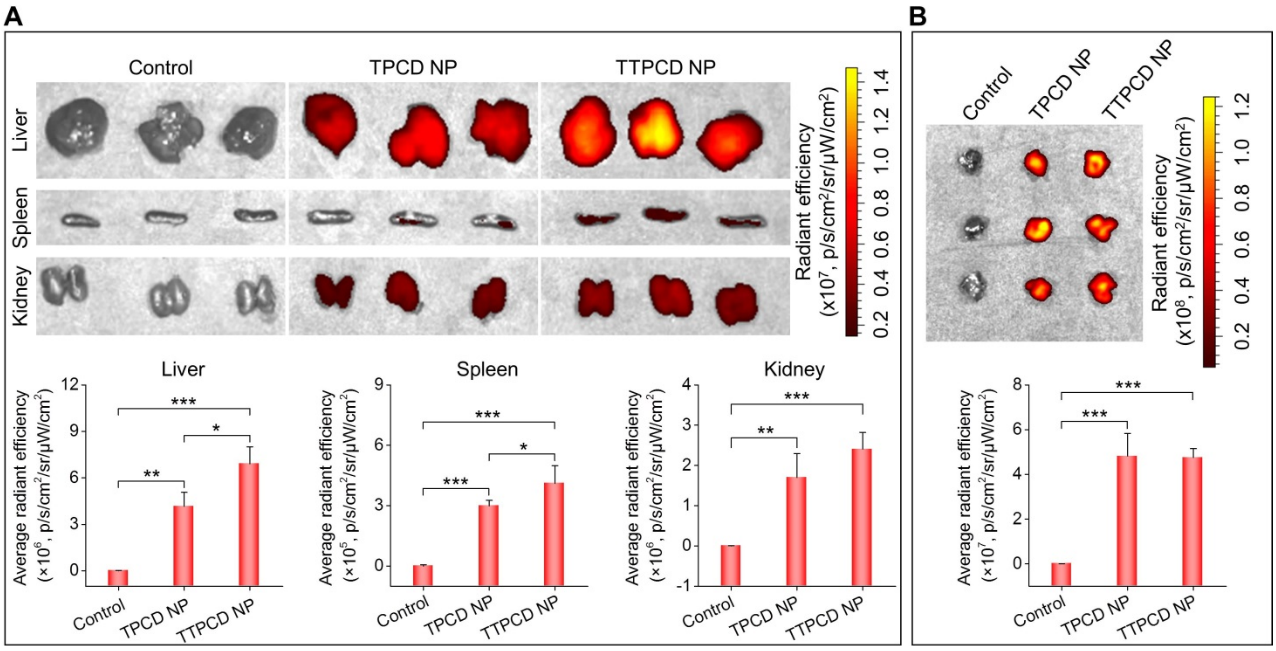

Figure S15. Accumulation of Cy7.5/TPCD NP or Cy7.5/TTPCD NP in major organs after inhalation. (A-B) *Ex vivo* fluorescence images and quantitative analysis show the distribution of Cy7.5/TPCD NP or Cy7.5/TTPCD NP in the liver, spleen, and kidneys (A) as well as in the lung (B) of mice after inhalation for 60 h. Data are mean ± SD (n = 3). *P < 0.05, **P < 0.01, ***P < 0.001.

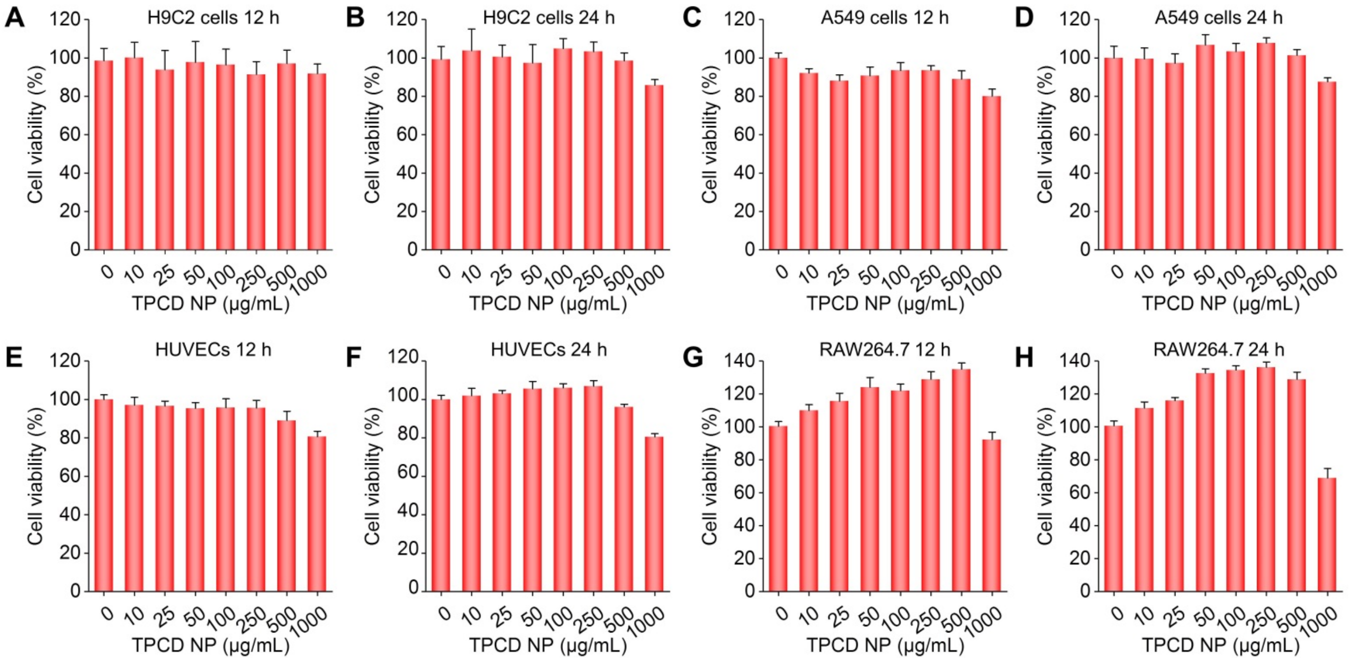

Figure S16. *In vitro* cytotoxicity evaluation of TPCD NP in different cells. (A-B) Cell viability values of H9C2 cells after incubation with various doses of TPCD NP for 12 h (A) or 24 h (B). (C-D) Cell viability values of A549 cells after incubation with various doses of TPCD NP for 12 h (C) or 24 h (D). (E-F) Cell viability values of HUVECs after incubation with various doses of TPCD NP for 12 h (E) or 24 h (F). (G-H) Cell viability values of RAW264.7 cells after incubation with various doses of TPCD NP for 12 h (G) or 24 h (H). A549 cells, HUVECs, and RAW264.7 cells were incubated at 1×10^4 cells per well, while H9C2 cells were at 5×10^3 per well. After cells were treated with different doses of TPCD NP for various time periods, cell viability was quantified by CCK-8 assay. Data are mean ± SD (n = 6).

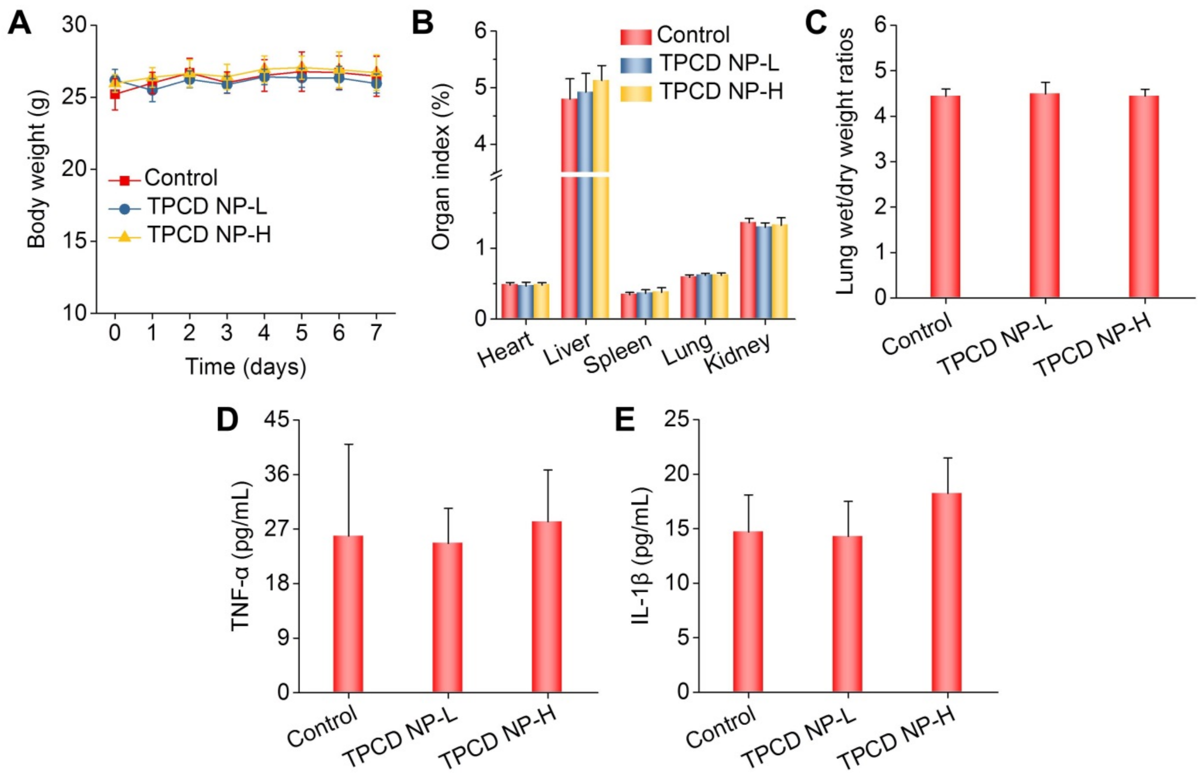

Figure S17. Acute toxicity evaluation of TPCD NP after inhalation at various doses for 7 days in mice. (A) Changes in the body weight of mice after inhalation of TPCD NP at 62.5 mg/kg (TPCD NP-L) or 125 mg/kg (TPCD NP-H). (B) The organ index values of mice after different treatments. (C) The lung wet/dry weight ratios after different treatments. (D-E) The expression levels of TNF-α (D) and IL-1β (E) in bronchoalveolar lavage fluid. In all cases, mice in the control group were treated by inhalation of saline. Data are mean ± SD (n = 5).

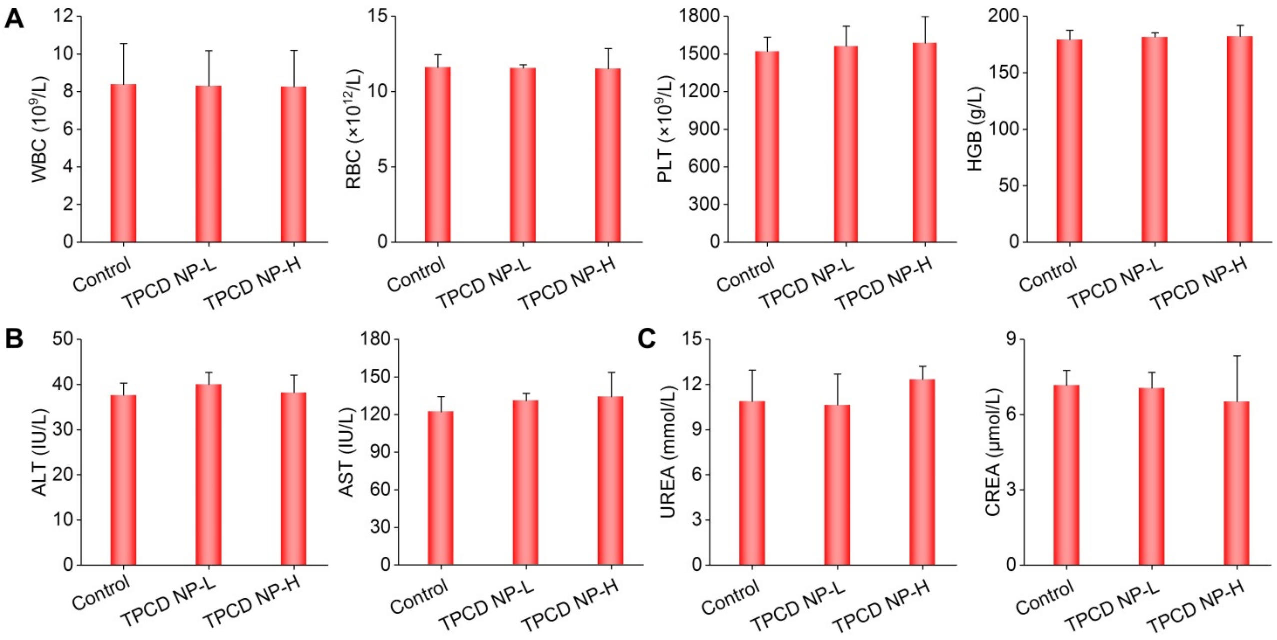

Figure S18. Levels of typical hematological parameters and biochemical markers of mice after acute toxicity tests. (A) The levels of WBC, RBC, PLT, and HGB. (B-C) Serum levels of biochemical indicators related to liver (B) and kidney (C) functions. WBC, white blood cells; RBC, red blood cells; PLT, platelets; HGB, hemoglobin; ALT, alanine aminotransferase; AST, aspartate aminotransferase; UREA, blood urea; CREA, creatinine. Data are mean ± SD (n = 5).

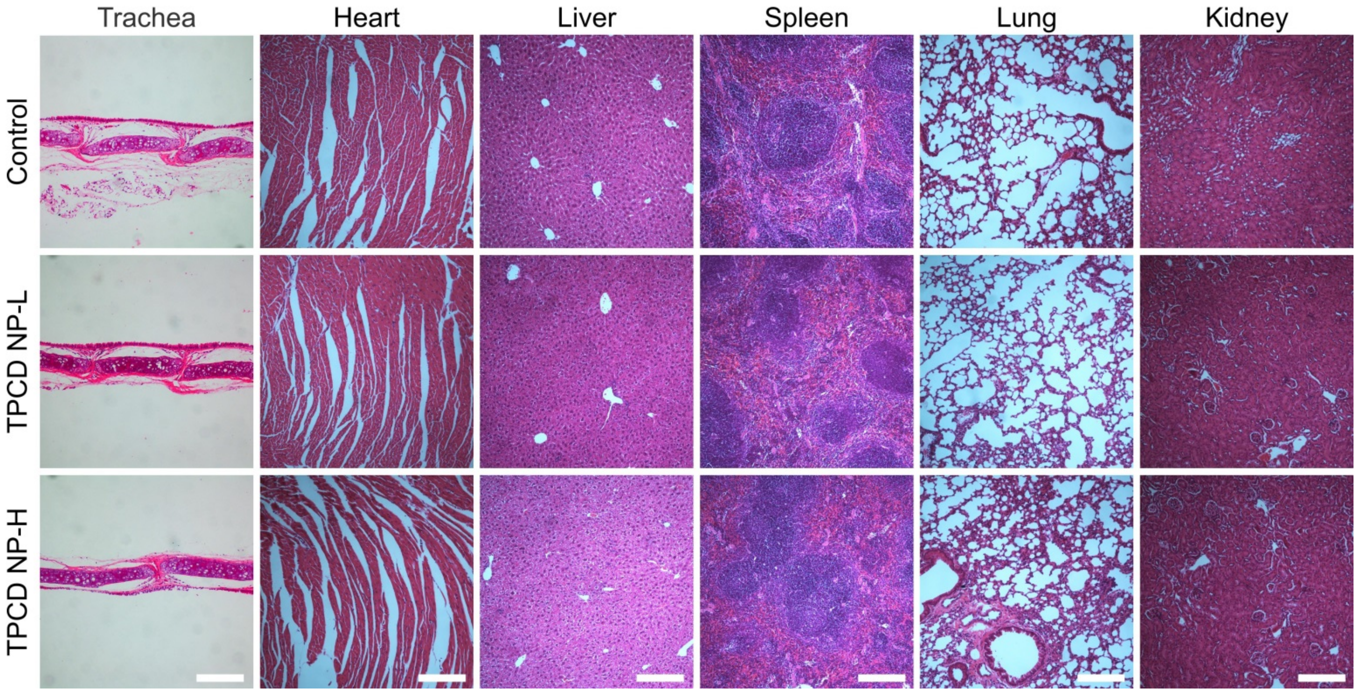

Figure S19. H&E-stained sections of major organs after different treatments. Scale bars, 200 µm.
